# Neogenin suppresses tumor progression and metastasis via inhibiting Merlin/YAP signaling

**DOI:** 10.1038/s41420-023-01345-w

**Published:** 2023-02-06

**Authors:** Xiaohan Hu, Li Li, Fang Li, Yuan Yang, Jingnan An, Xinghua Zhou, Rui Zhang, Lingli Shi, He Zhao, Jian Wang, Yizhou Hu, Yunyun Xu

**Affiliations:** 1 Pediatric Clinical Research Institute, Children’s Hospital Affiliated to Soochow University, Suzhou, Jiangsu China; 2 Department of Oncology, The Second Affiliated Hospital of Harbin Medical University, Harbin, Heilongjiang China; 3 Department of Human Anatomy, Histology and Embryology, School of Biology and Basic Medical Sciences, Soochow University, Suzhou, Jiangsu China; 4 Department of orthopedics, Honghui Hospital Affiliated to Xi’an Jiaotong University, Xi’an, Shaanxi China; 5 Institute of Blood and Marrow Transplantation, The First Affiliated Hospital of Soochow University, Suzhou, Jiangsu China; 6 Division of Molecular Neurobiology, Department of Medical Biochemistry and Biophysics, Karolinska Institute, Stockholm, Sweden

**Keywords:** Tumour-suppressor proteins, Metastasis, Prognostic markers

## Abstract

From in situ growth to invasive dissemination is the most lethal attribute of various tumor types. This transition is majorly mediated by the dynamic interplay between two cancer hallmarks, EMT and cell cycle. In this study, we applied nonlinear association analysis in 33 cancer types and found that most signaling receptors simultaneously associating with EMT and cell cycle are potential tumor suppressors. Here we find that a top co-associated receptor, Neogenin (NEO1), inhibits colorectal cancer (CRC) and Glioma in situ growth and metastasis by forming a complex with Merlin (NF2), and subsequent simultaneous promoting the phosphorylation of YAP. Furthermore, Neogenin protein level is associated with good prognosis and correlates with Merlin status in CRC and Glioma. Collectively, our results define Neogenin as a tumor suppressor in CRC and Glioma that acts by restricting oncogenic signaling by the Merlin-YAP pathway, and suggest Neogenin as a candidate therapeutic agent for CRC and Glioma.

## Introduction

The malignant initiation and the acquisition of invasive dissemination are two committed steps during tumor progression. Excessive mitotic activity is a hallmark of the initiation and in situ growth in most cancer types. Subsequently, these non-motile, polarized cancer cells acquire a series of genetic/epigenetic alterations and become invasive mesenchymal-like cells with life-threatening consequences [1]. A recent single-cell transcriptomics study uncovered that the cell cycle and epithelial-mesenchymal transition (EMT) are the most conserved functional gene programs across 22 cancer types [2]. During the tumor progression, these two functional programs tightly cooperate for tumor growth, invasion, and metastasis [3–5]. Therefore, the signaling pathway(s) that simultaneously conduct both programs can be a valuable molecular target for treating both in situ growing and disseminating tumors. However, these pathways, especially the receptors, have yet to be systematically elucidated.

In this study, we investigated the signaling receptor genes simultaneously associated with both cell cycle and EMT in a nonlinear manner among the bulk tissues of 33 tumor types. We identified many co-associated signaling receptors inversely co-correlated with EMT and cell cycle, indicating the importance of tumor suppressor genes during the tumor progression. We observed Neogenin, a canonical axon-guidance receptor, among the top inversely co-correlated receptors.

Neogenin appears to be a receptor or co-receptor for multi-ligands, including netrins, repulsive guidance molecules (RGM), and bone morphogenetic proteins (BMPs) [6–8]. Therefore, it has been implicated in various functions ranging from cell migration and survival to angiogenesis [9]. The expression of Neogenin is inversely correlated with malignancy of breast [10] and lung cancers [11], and loss of Neogenin expression is common in CRC [12]. However, the biological function of Neogenin in cancer cells and the underlying molecular mechanism are still unclear. Here, we experimentally validate the inhibitory effects of Neogenin during tumor initiation and dissemination in two different tumor models, Colorectal Cancer (CRC) and Glioma. We further revealed a previously unknown signaling axis that the association between Neogenin and Merlin directly modulates YAP activation in cancer cells.

## Results

### Neogenin inhibits tumor progression and is associated with good prognosis in CRC and glioma

**Fig. 1:**
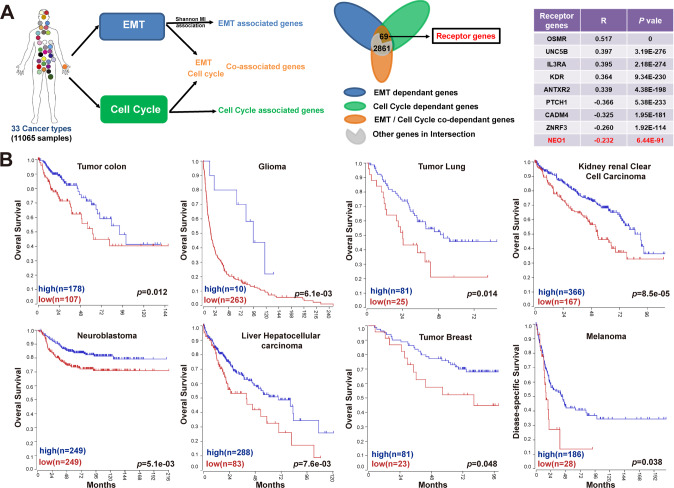
Enrichment of epithelial-mesenchymal transformation-related genes from 33 cancer types. **A** Gene expression data of 33 types of cancer were extracted from the TCGA dataset. The GO-bioprocess plot was generated by three major bio-functions: EMT, cell cycle, EMT and cell cycle process. As a result, 2930 genes significantly enriched in the intersection, with 69 receptor genes participating in the above three processes. The most significant positive and negative receptor genes were shown in the right table, and NEO1 was among them. **B** Overall Survival information and gene expression data of eight types of cancer patients were extracted from the TCGA dataset. The eight types of tumors involved are as follows: Tumor colon, glioma, tumor lung, kidney renal clear cell carcinoma, neuroblastoma, liver hepatocellular carcinoma, tumor breast, and melanoma. To describe the contribution of NEO1 to patient survival time, we used Kaplan–Meier analysis. 95% CI and *p* value were calculated via log-rank test and shown at the bottom of each plot.

We retrieved mRNA transcriptome sequencing data of 11,065 samples from 33 tumor types from the TCGA database. We calculated the EMT and mitotic scores for each tumor type via enrichment analysis. Next, to obtain the feature-associated gene sets in a nonlinear manner, we estimated the pairwise mutual information of each gene against the EMT score and the mitotic score, separately. Meanwhile, we performed the mutual information of triple variables for each gene against both the EMT score and the mitotic score. Among these three feature-associated gene sets, 2930 genes were significantly enriched in the intersection, with 69 receptor genes participating in the above three processes (Fig. 1A and Supplementary Table S4). Furthermore, the nonlinear monotonic association of these 69 receptors with the EMT score and mitotic score was analyzed by Spearman analysis, separately. Among the 69 receptor genes, most of the top EMT correlated receptor genes have been previously validated, such as OSMR, UNC5B, SEMA3F, and NOTCH2 [13–15], but rarely associated with the cell cycle in our association analysis. However, the top inversely correlated receptor genes with EMT also have negative regulatory relationships with mitotic activity, such as PTCH1, CADM4, ZNRF3, NEO1, etc. [16, 17] Here, we also found that NEO1 contributes significantly to EMT progress in 33 tumor models (Fig. 1A).

**Fig. 2:**
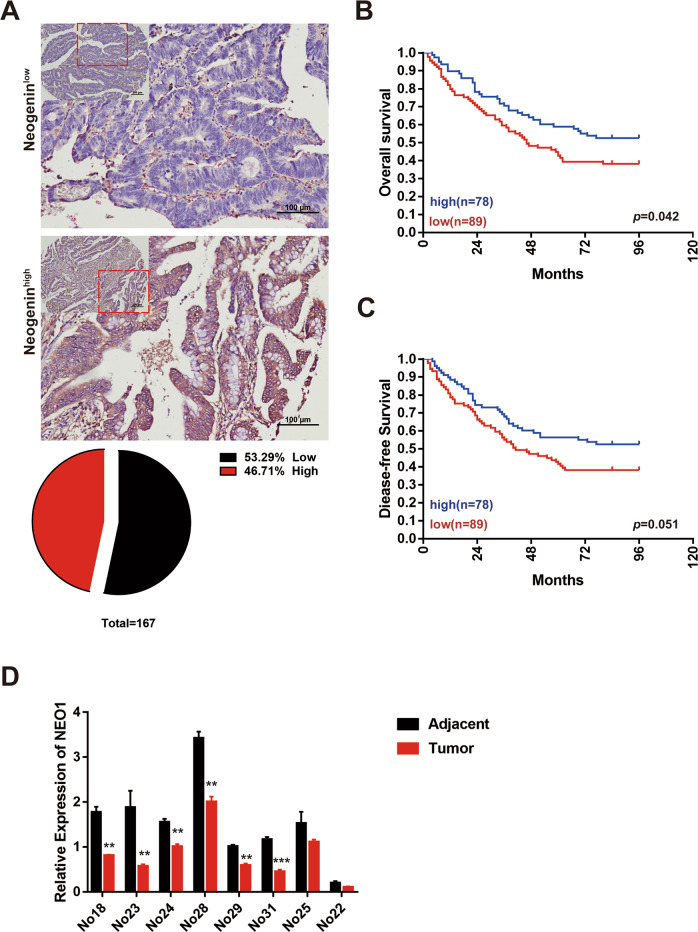
Neogenin is correlated with good prognosis in CRC and suppresses tumor growth. **A** IHC analysis of the expression of Neogenin in CRC tissues. **B**, **C** Kaplan–Meier plots of overall survival (**B**) and disease-free survival (**C**) of colorectal cancer patients. *p* value by log-rank tests. **D** Quantitative real-time PCR for NEO1 in resected CRC tumor tissue, and paired tumor-adjacent normal tissue (*n* = 8). Eight cases exhibit lower expression of NEO1 mRNA in tumor tissue. Data represent means ± SD. **p < 0.001, ***p < 0.0001. Significance determined by two-tailed Student’s *t*-test (**D**). See also Supplementary Fig. S1 and Supplementary Table S2.

To explore the role of NEO1 in cancer, we first analyzed the expression of Neogenin in eight tumor models, including tumor colon, glioma, tumor lung, kidney renal clear cell carcinoma, neuroblastoma, liver hepatocellular carcinoma, tumor breast, and melanoma, from TCGA and other public databases (Supplementary Table S5). Among all these tumor types, lower Neogenin expression predicts poorer overall survival (Fig. 1B). To further confirm the potential inhibitory function of Neogenin at the protein level, we collected 167 CRC biopsies and analyzed the protein expression of NEO1 by IHC (Fig. 2A and Supplementary Table S1). Among all cases, CRC patients with low expression of Neogenin have the poorest prognosis (Fig. 2B, C). Furthermore, we quantified the expression of NEO1 between tumor tissue and tumor-adjacent normal tissue in eight randomly selected CRC biopsies. Eight tumor tissues exhibited significantly lower expression of NEO1 at mRNA level (Fig. 2D). Moreover, we revealed that about 66.7% of glioma patients showed abnormally low expression of Neogenin protein via histological observation (Supplementary Fig. S1A). Besides, we accessed the expression of Neogenin protein in six CRC and two glioma cell lines. All cell lines harbored Neogenin protein at a low/undetected level (Supplementary Fig. S1B). Taken together, Neogenin is associated with good prognosis in both CRC and glioma.

### Neogenin suppresses the malignant behaviors of CRC and glioma cells

**Fig. 3:**
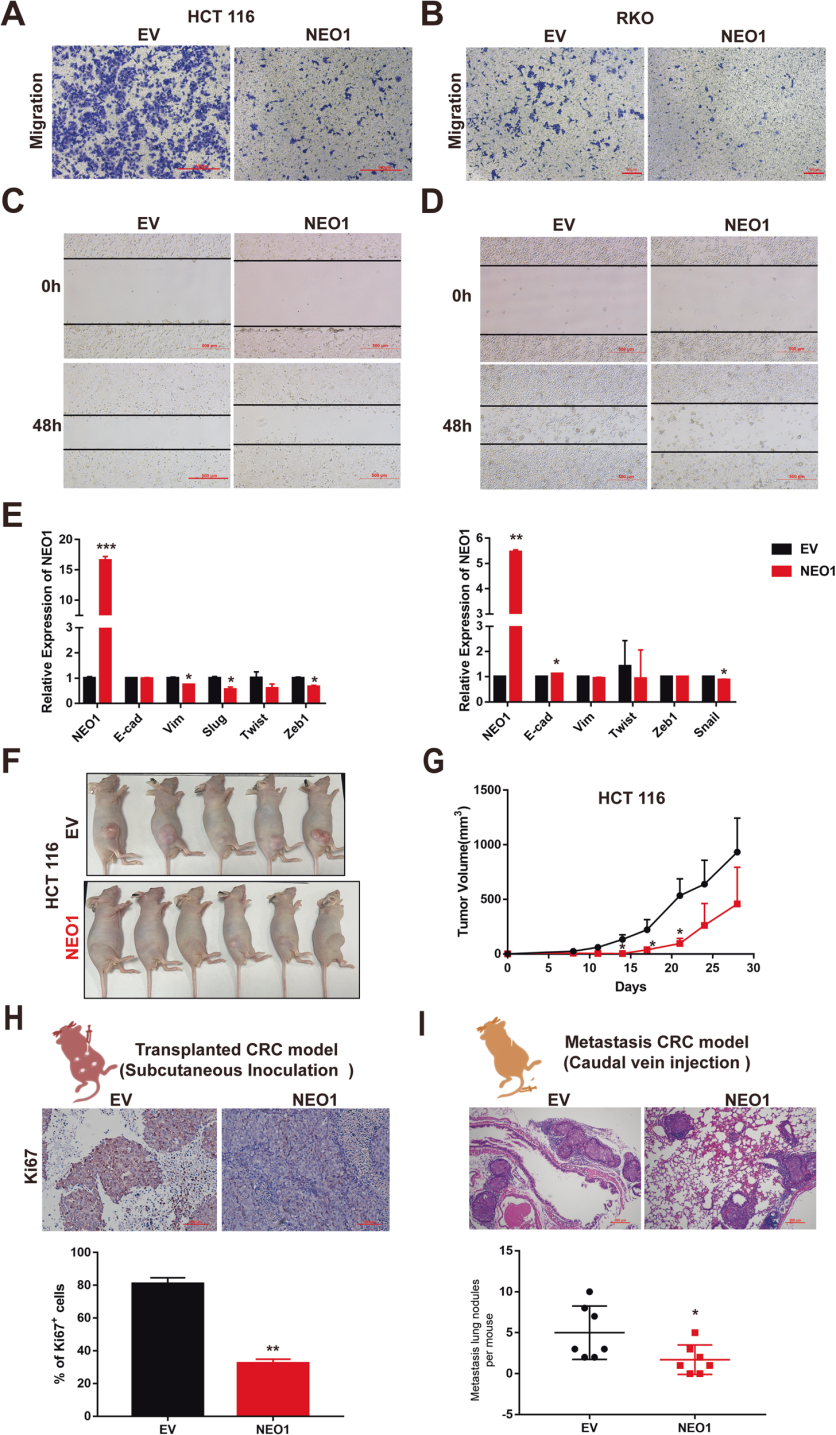
Neogenin suppresses the malignant behaviors of tumor cells. **A**–**D** NEO1 suppresses the motility of CRC cells. HCT 116 and RKO cells transduced with Lentivirus for NEO1 were applied for transwell assay (**A**, **B**) and scratch assay (**C**, **D**). Bar chat represents the area quantification of migrated cells. Scale bars, 100, 50, and 500 μm. **E** Quantitative RT-PCR for EMT-related genes and NEO1 in HCT 116 and RKO cells transduced with Lentivirus for NEO1. Gene names were listed at X-axis, and Y-axis represented the relative expression. **F** Representative images of tumor-bearing mice. Subcutaneous growth of control and NEO1-expressing HCT 116 cells in Nude mice. 5 × 10^6^ cells per injection, *n* ≥ 5 mice per group. **G** The primary tumor growth rate in different experimental groups is measured once per week. Experiments were performed in triplicate. **H** Primary tumor samples from the mice in Fig. 3F were collected for IHC staining of Ki67(up) and quantification of Ki67^+^ cells (down). *n* = 5 per group. Scale bars, 100 μm. **I** The control and NEO1-expressing HCT 116 cells were transplanted to Nude mice via the tail vein. 2 × 10^6^ cells per injection. H&E analysis metastatic nodule counts in the lung at the endpoint. *n* = 5 lungs per group. Scale bars, 200 μm. Data represent means ± SD. *p < 0.05, **p < 0.001, ***p < 0.0001. Significance determined by two-tailed Student’s *t*-test (**E**–**I**). See also Supplementary Figs. S2–S4.

To directly test the putative tumor-suppressive functions of Neogenin, we used a lentiviral vector to stably overexpress human NEO1 in the CRC and Glioma cell lines with low NEO1 expression levels (Supplementary Fig. S2A). NEO1 overexpression attenuated Vimentin in HCT 116 CRC cells and U87MG Glioma cells at the protein level, respectively (Supplementary Fig. S2B, C). To further confirm the result, a set of tumor-suppressive functions regulated by NEO1 were observed in CRC or glioma cells. Cells with enhanced NEO1 expression exhibited an impaired ability of migration and invasion in two CRC cell lines (HCT 116, RKO) (Fig. 3A–D) and two Glioma cell lines (U87MG, U251) (Supplementary Fig. S4A–D). In contrast, CRC SW480 Cells with downregulated NEO1 expression exhibited an enhanced ability to migrate (Supplementary Fig. S3). Meanwhile, in all these four cell lines, the mRNA expression of EMT-associated markers changed markedly and exhibited attenuated mesenchymal-like features after NEO1 overexpression (Fig. 3E and Supplementary Fig S4E). Moreover, NEO1-expressing CRC HCT 116 cells are rarely able to form tumors in vivo. The tumor formation of the human HCT 116 cells emerged after 4 weeks, while delayed tumorgenicity and smaller tumor size were observed in NEO1-overexpressing group (Fig. 3F, G). Similarly, the tumorigenicity of NEO1-expressing U87MG cells was significantly reduced in vivo (Supplementary Fig. S4F, G). Primary tumors from the NEO1 gene overexpressing group had significantly fewer Ki67^+^ cells (Fig. 3H and Supplementary Fig. S4H). In addition, to further evaluate the therapeutic potential of NEO1 treatment during metastasis, NEO1-expressing or control CRC HCT 116 cells were injected intravenously to generate lung metastasis. As a result, NEO1 overexpression significantly reduced metastatic growth (Fig. 3I). Collectively, these data suggested that Neogenin has robust efficacy in inhibiting in situ growth and metastasis.

### Identification of Neogenin-interacting protein Merlin

**Fig. 4:**
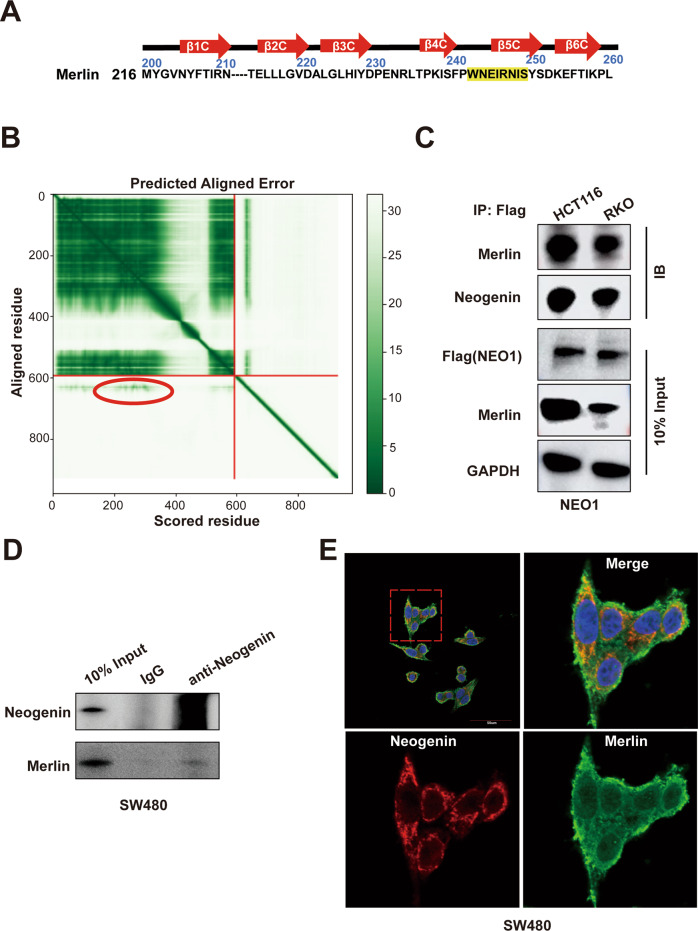
Identification of Neogenin-interacting protein Merlin. **A** Neogenin interacts with the β5C strand and α1C helix of FERM subdomain C of Merlin (A.a.: WNEIRNIS). **B** Alpha fold multimer prediction. **C** HCT 116 and RKO cells stably expressing NEO1-Flag were lysed and IP with IgG or anti-Flag antibodies. The IP samples were subjected to WB analysis with the interaction between Neogenin and Merlin. **D** The interaction of Neogenin and Merlin in SW480 was validated by immunoprecipitation assay. Cell lysates from SW480 were IP against Neogenin and IB against Merlin. Merlin protein was pulled down with an anti-Neogenin antibody, but not IgG. **E** SW480 cells were cultured with a normal medium for 24 h for immunostaining of Neogenin (red) and Merlin (green). Both Neogenin aggregates co-localize with Merlin at the cell edge and cytoplasm region. Scale bars, 10 µm. See also Supplementary Fig. S5.

We next investigated the underlying mechanism of NEO1-induced suppression of tumor growth. The homolog of NEO1 [18], DCC, is a docking protein for many molecules containing the FERM domain, such as myosin X, Ezrin, etc. [19, 20]. This docking interaction depends on the P3 domain of the DCC tail, and the β5C strand and α1C helix of FERM subdomain C [21]. Interestingly, the P3 domain is evolutionarily conserved between DCC and Neogenin [19], and the β5C strand and α1C helix of FERM subdomain C are shared by various types of FERM proteins [22] (Fig. 4A). Therefore, we assume that Merlin (NF2), a well-defined tumor suppressor with FERM domain [23], can bind to Neogenin and mediates its inhibitory effects in tumor cells. To validate this, we first predicted the Neogenin-Merlin protein complex via Alpha Fold Multimer prediction (Supplementary Fig. S5). The prediction indicated that Merlin’s most proper binding site to the Neogenin tail is the subdomain C at the FERM region, as highlighted with the red circle in the left-bottom area (Fig. 4B). Furthermore, the second structure visualization of the Merlin/Neogenin interaction suggested that Merlin’s most proper binding site is the β5C region. Next, we validated the interaction between Neogenin and Merlin by immunoprecipitation of CRC HCT 116 and RKO cells stably expressing NEO1-Flag (Fig. 4C). Moreover, the endogenous interaction was confirmed using CRC SW480 cells with high endogenous expression of NEO1 (Fig. 4D). In addition, immunofluorescence assay in SW480 cells (Fig. 4E) revealed that Neogenin and Merlin co-localized at the cell edge. Thus, these findings revealed that Merlin is an interacting partner for Neogenin.

### Neogenin/Merlin modulate EMT to inhibit tumor cell malignancy

**Fig. 5:**
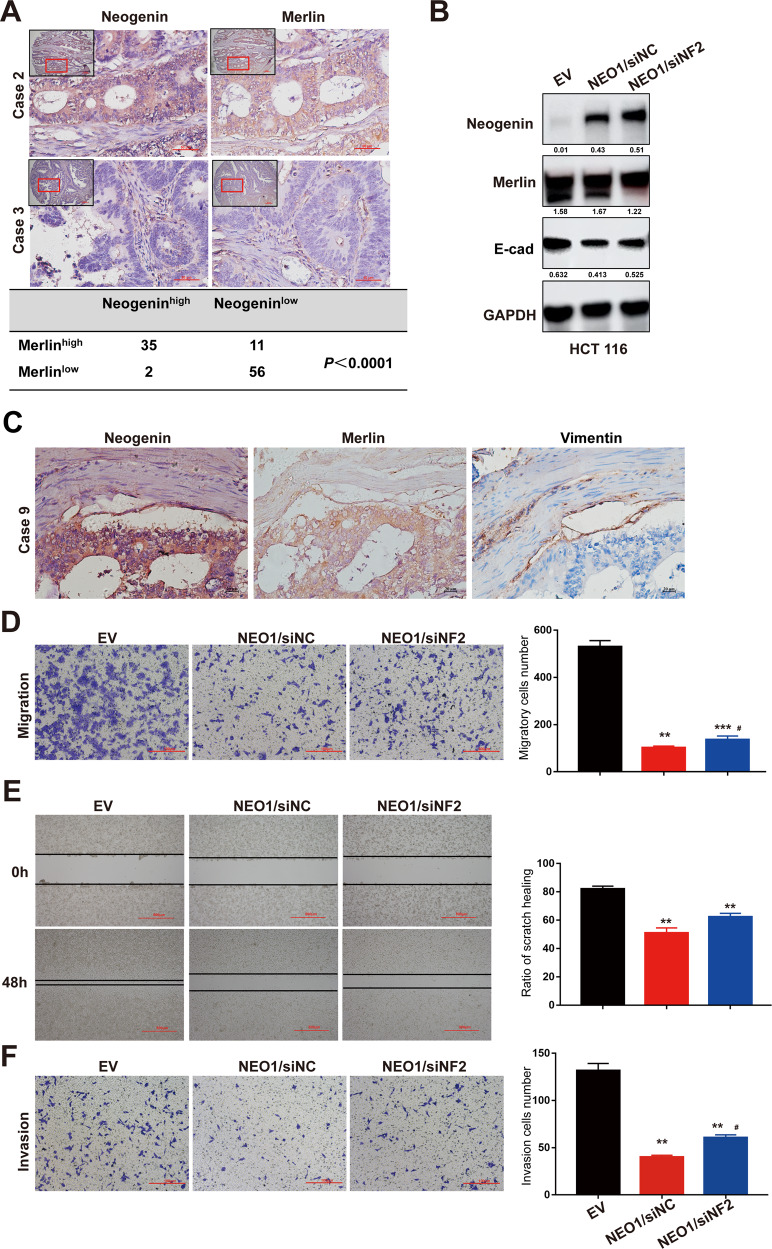
Neogenin/Merlin modulates EMT to inhibit tumor cell malignancy. **A** Representative image of IHC staining (up) and the correlation between Neogenin and Merlin (down) in primary tumors from CRC patients. Scale bars 50 μm. **B** NEO1 downregulates the expression of E-cad in CRC cells, but interfering with NF2 expression reverses the inhibition. HCT 116 cells transduced with lentivirus for NEO1 and siRNA for NF2 was applied for WB assay. siNC was used as control. **C** Representative IHC staining of Neogenin, Merlin, and vimentin in primary tumors from CRC patients. Scale bars 20 μm. **D**–**F** NEO1 suppresses the motility of CRC cells, but interfering with NF2 expression reverses the inhibitory effect. HCT 116 cells transduced with lentivirus for NEO1 and siRNA for NF2 was applied for migration assay (**D**, **E**) and invasion assay (**F**). siNC was used as control. The bar chart represents the quantification of migration assay (**D**, **E**) and invasion assay (**F**). Data represent means ± SD. *p < 0.05, **p < 0.001, ***p < 0.0001 compared with EV group by multiple *t*-tests or two-tailed Student’s *t*-test (**D**–**F**). ^#^*p* < 0.05, ^##^*p* < 0.001, ^###^*p* < 0^.^0001 compared with NEO1/siNC group. See also Supplementary Fig. S6.

IHC staining of the primary tumors in CRC (Fig. 5A) and Glioma patients (Supplementary Fig. S6A) indicated that Neogenin protein amounts correlated with Merlin protein. Importantly, we found that EMT-related Vimentin showed low expression when both Neogenin and Merlin were highly expressed in tumor tissues from the CRC patient (Fig. 5C). Consistently, silencing Merlin partially rescued the expression change of EMT-related marker genes in NEO1-overexpressing cells (Fig. 5B and Supplementary Fig. S6B). Furthermore, NEO1 overexpression inhibited the motility of CRC cells (HCT 116) and glioma cells (U251), and this inhibition was partially abolished after silencing NF2 (Fig. 5D, E and Supplementary Fig. S6C, D). Similar findings were observed in the invasion assay of CRC cells (HCT 116) and glioma cells (U87MG) (Fig. 5F and Supplementary Fig. S6E). Together, our data demonstrated that Neogenin interacts with Merlin to inhibit tumor malignancy by obstructing EMT progress, and the lack of Neogenin/Merlin is associated with the poorest prognosis in CRC and glioma patients.

### Neogenin exerts its tumor-suppressive function by Merlin/YAP Signaling pathway

**Fig. 6:**
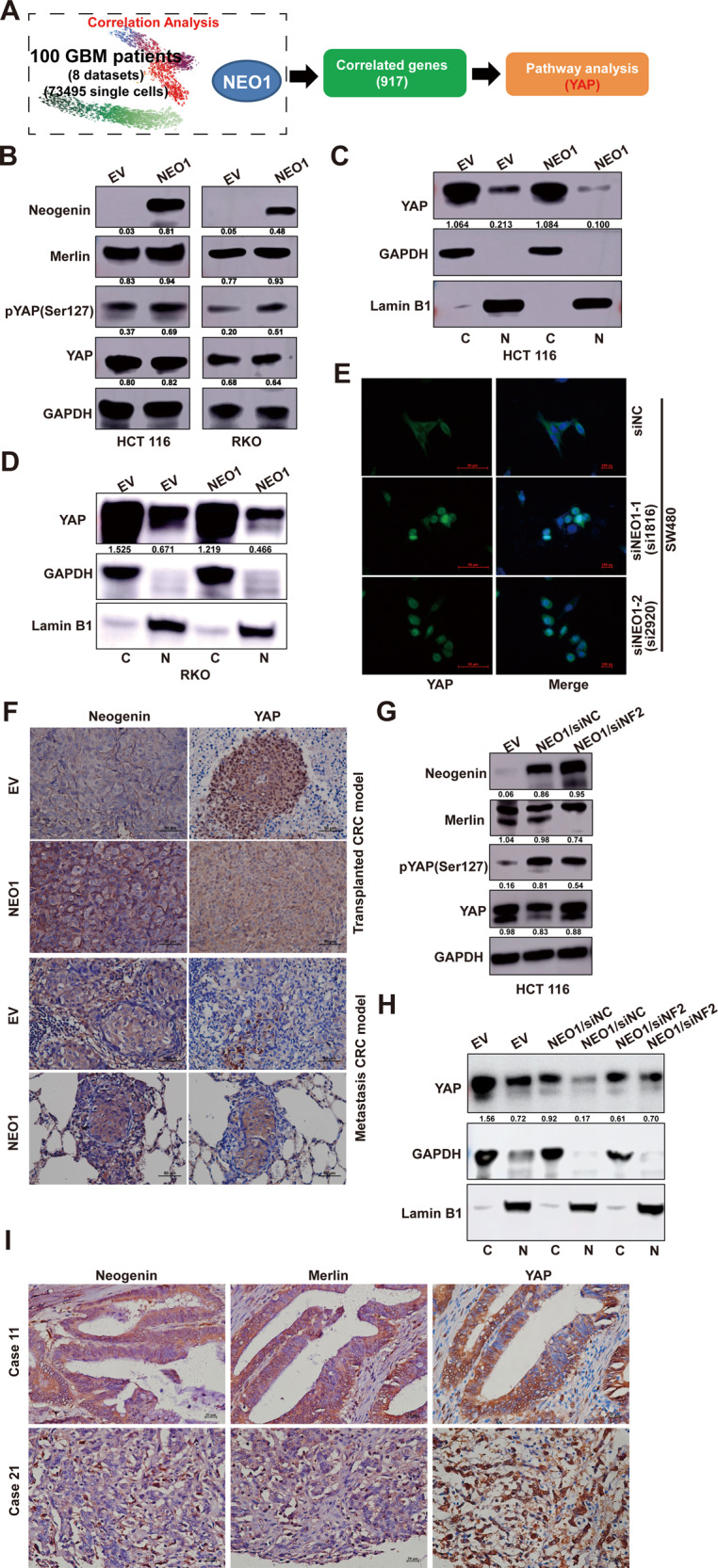
Neogenin exerts its tumor-suppressive function by Merlin/YAP signaling Pathways. **A** The single-cell mRNA transcriptome sequencing data of 100 GBM patients was extracted from eight TCGA datasets. We enriched 917 essential genes closely related to NEO1 by bioinformatic clustering analysis, and then found YAP signal by pathway analysis. **B** The phosphorylation of YAP was activated after the expression of NEO1. NEO1 represents the overexpression of NEO1 in CRC cells, and EV was used as control. The protein levels of Neogenin, Merlin, YAP, and phosphorylation of YAP in HCT 116 and RKO cells were determined by immunoblotting. **C**, **D** The subcellular fractionation analysis of YAP expression in HCT 116 and RKO cells stably expressing NEO1 (NEO1) or control (EV). Immunoblotting of GAPDH and Lamin B1 served as controls for the purity of cytoplasmic (C) and nuclear (N) fractions, respectively. **E** Immunofluorescent staining of YAP (Green) in SW480 cells stably transduced with siRNA for NEO1(si1816, si2920), and siNC was non-targeting control. Nuclei were stained with DAPI (blue). Scale bars 50 μm. **F** Representative images of IHC staining of Neogenin and YAP in tumors from transplanted CRC and metastasis CRC model. Scale bars 50 μm. **G** NEO1 promotes the phosphorylation of YAP in CRC cells, but interfering with NF2 expression reverses the activated effect. HCT 116 cells transduced with Lentivirus for NEO1 and siRNA for NF2 was applied for WB assay. siNC was used as control. **H** The subcellular fractionation analysis of YAP expression in HCT 116 cells from Fig. 6G. Immunoblotting of GAPDH and Lamin B1 served as controls for the purity of cytoplasmic (C) and nuclear (N) fractions, respectively. **I** Representative IHC staining of Neogenin, Merlin, and YAP in primary tumors from CRC patients. Scale bars 20 μm. See also Supplementary Fig. S7.

To explore the downstream signaling pathway mediated by NEO1/NF2, we retrieved single-cell mRNA transcriptome sequencing data of 100 GBM patients from eight datasets and performed nonlinear monotonic spearman correlation of NEO1 against all other genes for each dataset. Nine hundred seventeen conserved NEO1-associated genes were selected with the standard that the correlation occurs in at least two datasets. Subsequent pathway analysis enriched the YAP-TAZ pathway (Fig. 6A and Supplementary Table S6). Considering that the Caherlin-NF2-Hippo-YAP signaling axis frequently appears in tumors [24], we can draw the following inference: Neogenin/Merlin is involved in tumor progression by regulating the signaling molecule YAP.

**Fig. 7:**
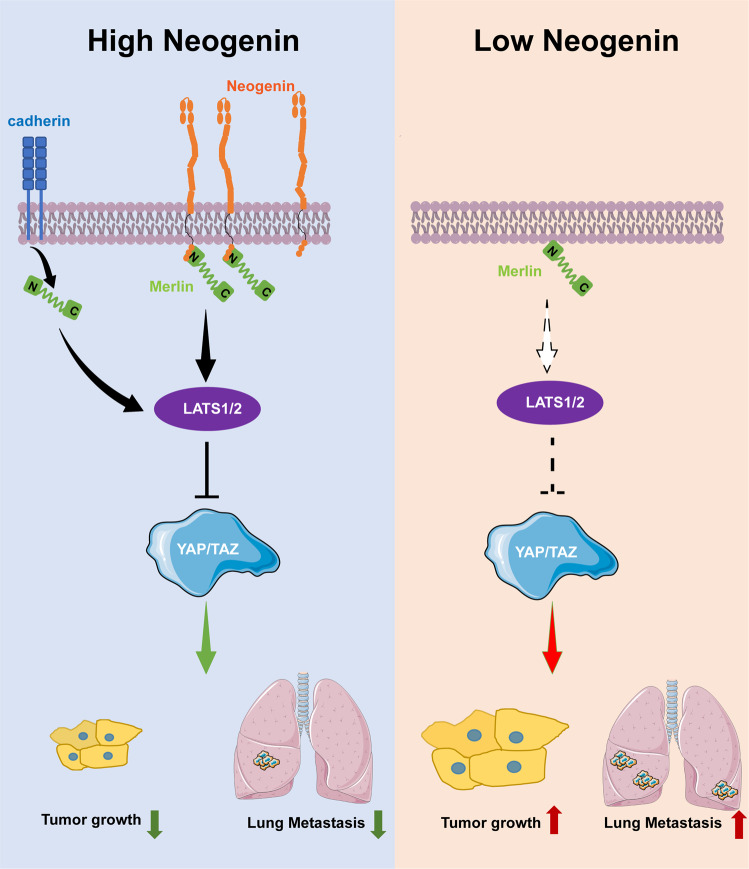
Model of Neogenin regulation of tumorigenesis and metastasis. Schematic illustration depicting the roles of Neogenin in suppressing tumor growth and metastasis. Note that the anti-oncogenic potential of Neogenin is likely attributed to Merlin/YAP pathway.

To experimentally test this hypothesis, we assessed the effects of NEO1 on YAP signaling, and we found that YAP phosphorylation significantly increased after the overexpression of NEO1 in CRC and Glioma cells (Fig. 6B and Supplementary Fig. S7A). YAP phosphorylation causes cytoplasmic retention, thereby preventing its function as a transcriptional coactivator in the nucleus [25]. In line with this observation, overexpression of NEO1 in CRC HCT 116, RKO, and glioma U251 cells significantly reduced the levels of nuclear YAP (Fig. 6C, D and Supplementary Fig. S7B). Silencing NEO1 in SW480 cells shifted the main location of YAP from the cytoplasm to the nucleus (Fig. 6E). In the primary tumors and lung lesions of HCT 116 and U87MG cells inoculation, NEO1 overexpression reduced the expression of nuclear YAP (Fig. 6F and Supplementary Fig S7C). Furthermore, NEO1-induced pYAP activation was dramatically attenuated by silencing NF2 in both CRC and Glioma cells (Fig. 6G and Supplementary Fig. S7D). Consistently, NEO1-reduced YAP expression in the nucleus was markedly reversed by silencing NF2 in CRC HCT 116 cells (Fig. 6H). More importantly, we also found that CRC patients with low expression of Neogenin and Merlin had tumor cells in which the YAP protein was mainly expressed in the nucleus (Fig. 6I). In summary, these findings suggest that Neogenin regulation of Merlin/YAP plays a functional role in regulating the disease progression of CRC and glioma (Fig. 7).

## Discussion

From in situ growth to invasive dissemination is the most lethal attribute of various types of tumors, and it is majorly mediated by the dynamic interplay between two cancer hallmarks, EMT and cell cycle [26–28]. In this study, we applied nonlinear association analysis in 33 cancer types and found that most signaling receptors simultaneously associating with EMT and cell cycle are potential tumor suppressors. Furthermore, our in vitro and in vivo experiments validated the inhibitory effect of a top co-associated receptor, Neogenin. And we uncovered its underlying novel signaling axis of NEO1-NF2-YAP in CRC and Glioma.

Accumulating studies have shown that EMT is a critical start-up step for regulating tumor metastasis [26–33]. We found that the top EMT correlated receptors rarely or even inversely associated with the cell cycle. This finding is in line with previous reports that several transcription factors, such as Twist, Snail, and Slug, can induce EMT and simultaneously promote prolonged cell cycle arrest [34–36]. However, the top receptors with reduced expression during EMT usually exhibited less cell cycle activity. This finding suggests that the dysfunction of tumor suppressor receptors is a general condition for both in situ growth and dissemination during tumor progression.

Significantly, CRC patients with low Neogenin expression levels have shorter survival time than those with high Neogenin expression levels. Consistently, the patient prognosis in ten types of tumors negatively correlates with Neogenin by analyzing TCGA Databases. In addition, the functional studies show that silencing of Neogenin drove CRC cell growth, invasion, and metastatic properties both in vitro and in vivo. Mechanistically, Neogenin suppresses CRC and Glioma progression and metastasis through binding to Merlin and subsequently promotes YAP phosphorylation. Together, our study confirms Neogenin as a suppressor of tumor growth and metastasis in CRC and Glioma.

Neogenin, DCC paralogue, shares ~50% amino acid identity with DCC [12]. Evolutional evidence suggests that Neogenin is possibly more conserved than its paralogue-DCC for two reasons: first, Neogenin contains more alternatively spliced regions; second, some species lack DCC while expressing Neogenin. Only the NEO1 gene has been found in chickens but not the DCC gene [37]. In zebrafish, DCC and NEO1 are two independent genes, while the expression pattern of NEO1 during embryogenesis is more extensive than that of DCC [38, 39]. The intracellular tail region of DCC/Neogenin consists of three evolutionally conserved domains termed P1, P2, and P3 motifs [40]. The original axon-guidance function of Neogenin/DCC is majorly mediated and regulated by three pathways: (1) influx of calcium ion [41]; (2) cyclic nucleotides (cAMP or cGMP) and protein kinase A (PKA) [42]; (3) the activity of Rac and Rho [43]. In addition, a recent study revealed a new signaling pathway of Neogenin/RhoA/YAP/Smad1 involving the astrocyte differentiation, indicating the Hippo pathway as a downstream signaling target of Neogenin [44].

Except for the gastric cancer cells [33, 34], Neogenin has been indicated to be a tumor suppressor in different cancer types, such as breast cancer [10], glioblastoma [45], and lung cancers [11]. In line with these findings, our results show that, in our analyzed eight types of tumors, low expression of Neogenin predicts worse patient prognosis (Fig. 1B), and we found that Neogenin has robust efficacy in inhibiting the outgrowth, migration, and invasion of CRC or glioma cells. Especially, forced expression of Neogenin significantly reduces in situ growth and metastasis in CRC in vivo. Thus, we computationally and experimentally validated that Neogenin functions as a general suppressive receptor in various types of cancer. However, how Neogenin suppresses tumor progression remains unclear.

Here, we found that Merlin (NF2) associated and transduced the inhibitory signaling of Neogenin via YAP in cancer cells. Merlin was a canonical tumor suppressor protein encoded by the neurofibromatosis type 2 gene, *NF2* [23]. Loss-of-function mutations in NF2 lead to tumorigenesis, including schwannoma, meningioma, and ependymoma [46–48]. In a very aggressive brain tumor GBM, Merlin protein and mRNA levels were decreased by over 50% in GBM compared to normal human astrocytes and brain tissue [49]. In addition, Merlin showed antitumor activity and increased apoptosis in multiple tumors, including CRC [50], lung squamous cell carcinoma [51], osteosarcoma [52], and pancreatic cancer [53].

Structurally, Merlin is highly related to the cytoskeleton and membrane-linking proteins, including the ERM proteins (ezrin, radixin, and myosin) and myosin X protein. Among all these proteins, the β5C strand and α1C helix of FERM subdomain C are evolutionarily conserved [22]. These two epitopes have been shown as the docking sites for the DCC tail, which is also evolutionarily shared with Neogenin [21]. In light of this evidence, we in silico predicted and experimentally validated the interaction between Merlin and Neogenin in CRC and Glioma cells.

Merlin regulated multiple signaling pathways and integrated extracellular signals. Merlin signaling pathways lead to tumorigenesis, tumor progression, and pharmacological resistance via different molecular mechanisms [23]. Merlin’s most crucial mechanism was the regulation of the canonical hippo pathway. In the canonical hippo pathway, mammalian Ste20-like kinases (MST1/2) phosphorylate large tumor suppressor kinases (LATS1/2). LATS1/2, in turn, phosphorylates and inactivates YAP/TAZ, thus blocking TEAD/MEAD transcription factor [54]. Besides the canonical hippo pathway, Yap activity can be regulated by alternative pathways, such as cell mechano-transduction, Rho-GTPase signaling, inflammation, and G-protein coupled receptor (GPCR) signaling [55–58]. However, recent findings indicated that the canonical Hippo pathway, especially the LATS1/2, may function as a reinforcing mechanism and require other alternative pathways to fully modulate YAP/TAZ-TEAD activity [59–61].

Our results suggested that Neogenin showed antitumor activity via the canonical signaling cascade of Merlin- MST1/2-LATS1/2-YAP. Previous findings show that Neogenin participated in the alternative YAP signaling via RhoA. And ERM proteins, including merlin, exhibited signaling crosslink with RhoA [62, 63]. Thus, we proposed that Neogenin suppressed tumor progression via targeting YAP signaling in both the canonical Hippo-dependent and -independent manner.

In summary, our in silico and experimental studies indicated that Neogenin might suppress different cancer types via targeting YAP signaling, which was mediated via the newly identified interactor of Merlin. Future studies should explore the potential therapeutic benefit of Neogenin in diverse cancer types.

## Materials and methods

### Patients and clinical samples

Expression levels of Neogenin and Merlin were measured using the immunohistochemical assay in 167 patients with CRC who underwent the operation and were confirmed by pathology at the First Affiliated Hospital, Soochow University (Suzhou, Jiangsu, P.R.C) between 2004 and 2007. The clinicopathologic characteristics of all patients were described in Supplementary Material (Supplementary Table S1). Additionally, eight fresh tissues from patients, confirmed as CRC at the same hospital, were used to analyze the expression of NEO1 mRNA and protein level. Our study was approved by the First Hospital Affiliated to Soochow University for Biomedical Research Ethics Committee, and all patients provided informed consent.

### Cell lines

The CRC cell lines HCT 116 and RKO were obtained from the Cell Bank of the Chinese Academy of Sciences (Shanghai, P.R.C), which performs routine cell line authentication testing with SNP and short tandem repeat analyses. The other four CRC cell lines (HT-29, DLD-1, SW480, and SW620) were obtained from the International Joint Cancer Institute, The Second Military Medical University (Shanghai, P.R.C). The Glioma cell lines U251 and U87MG were purchased from the Procell Life Science&Technology Co., Ltd., which verifies the authenticity of cells by STR profiling. All the cell lines were cultured according to the supplier’s instructions, and were used in the culture at the fifth through tenth passage for this study.

### Generation of the cell line for inducible NEO1 overexpression or Merlin downregulation

HCT 116 and RKO cells were transfected with the lentiviral vectors encoding the human NEO1 gene, which were generated by using the GV341-puro vector and designated as NEO1. The empty vector was used as a negative control, and was designated as EV. All the Lentiviral vectors used in this study were synthesized by Genechem (Shanghai, P.R.C). Furthermore, HCT 116 and RKO cells stably expressing NEO1 (NEO1) or negative control (EV) were reconstructed using a siRNA technique (GenePharma, Shanghai, P.R.C). The siRNA targeting specific human Merlin sites (si774 and si1952) and negative control siRNA (siNC) were shown in the Supplementary Material (Supplementary Table S2). The overexpression of NEO1 and downregulation of Merlin were validated by Quantitative RT-PCR and western blot analysis. In addition, SW480 cells of highly-expressed NEO1 were downregulated by siRNA targeting specific human NEO1 sites (si1816 and si2920), as shown in the Supplementary Material (Supplementary Table S2).

### Quantitative RT-PCR analysis

Total RNA was isolated using by using the NucleoSpin RNA kit (#740955, MACHEREY-NAGEL, Duren, Germany). Then the first-strand cDNA was generated by using the HiScript III 1st Strand cDNA Synthesis kit (#R312, Vazyme, Nanjing, P.R.C). The quantitative reverse transcription PCR (qRT-PCR) was performed by using the ChamQb SYBR qPCR Master Mix kit (#Q311, Vazyme, Nanjing, P.R.C) according to the manufacturer’s instruction. Data were collected and analyzed with a CFX Connect Real-Time PCR Detection System instrument (Roche lightcycler480 II, CA, USA). The mRNA expression was normalized by the expression of β-actin. The primer sequences were listed in the Supplementary Material (Supplementary Table S3).

### Western blotting (WB) analysis

Nuclear and non-nuclear (membranes and cytosol) fractions were prepared using the Nuclear and Cytoplasmic Protein Extraction Kit (Beyotime Biotechnology, Shanghai, P.R.C). Protein was extracted from the cells were resolved by SDS-PAGE and then transferred to PVDF membranes (0.45 μm, Millipore, Billerica, MA, USA), and then incubated with primary antibodies diluted in blocking buffer at 4 °C overnight. The following primary antibodies were used: rabbit anti-Neogenin (Abcam, Cambridge, MA, USA), mouse anti-Merlin (Novus biologicals, USA), rabbit anti-Merlin, rabbit anti-phospho-YAP (Ser127), rabbit anti-YAP (Cell Signaling Technology, Danvers, MA, USA), mouse anti-Neogenin (Santa Cruz Biotechnology, MA, USA), mouse anti-β-actin, mouse anti-Flag, rabbit anti-Flag (Sigma Aldrich). Horseradish peroxidase (HRP) conjugated secondary antibody (Anti-rabbit IgG, HRP-linked Antibody #7074; Anti-mouse IgG, HRP-linked Antibody #7076, Cell Signaling Technology) was used. Finally, the antigen-antibody reaction was visualized by the enhanced Pierce ECL Western blotting substrate kit (Thermo Scientific/Pierce, Rockford, IL, USA).

### Immunoprecipitation (IP) analysis

For the IP experiment, Cells were collected in cell lysis buffer (Beyotime Biotechnology) supplemented with the Protease inhibitor cocktail (Beyotime Biotechnology). The cell lysates were immunoprecipitated with mouse anti-Flag antibody (1:50, Sigma Aldrich) overnight, followed by incubation with Protein A/G Magnetic Beads (Bimake, USA) for 2 h. Finally, immunoprecipitated protein complexes were detected using WB.

Furthermore, the cell lysate of CRC SW480 cells was immunoprecipitated with mouse anti-Neogenin antibody (2 μg/200 μL supernatant, Santa Cruz Biotechnology), separated by SDS-PAGE and subjected to WB analysis with rabbit anti-Merlin antibody.

### Immunofluorescence

CRC cells were seeded on coverslips in 24-well plates overnight, and then were fixed, permeabilized, and blocked. After blocking, cells were incubated with primary antibodies specific for rabbit anti-Merlin, rabbit anti-YAP (Cell Signaling Technology), and mouse anti-Neogenin (Santa Cruz Biotechnology) overnight at 4 °C. Moreover, incubation of HRP-conjugated secondary antibodies (Life Technologies, Duren, DE) and TSA^TM^ System (#2336651, Fluorescein System; #2384212, Cyanine 3 Syaytem, PerkinElmer, MA, USA) were separately carried out for 30 and 10 min at room temperature. Finally, DAPI (Dojindo Laboratories, Kumamoto, Japan) was then used for counterstaining the nuclei and images were obtained by laser scanning confocal microscopy (OLYMPUS IX83, Tokyo, Japan).

### Immunohistochemistry (IHC) staining

Paraffin-embedded primary tumor tissues or lung samples were sliced into 4-μm thicknesses. To remove aldehyde links formed during the initial fixation of tissues, antigen retrieval had to be performed by a pressure cooker for 3 min in 0.01 M citrate buffer (pH 6.0). Moreover, histological sections were separately incubated with specific antibodies for Neogenin (1:100, Proteintech, Hubei, P.R.C), Merlin (1:100, Cell Signaling Technology), and YAP (1:400, Cell Signaling Technology) overnight at 4 °C. The immunodetection was performed on the following day using DAB (Dako, Carpinteria, CA) according to the manufacturer’s instructions. Finally, immunostaining scores were evaluated as described previously [64] (Supplementary Table S1).

### Cell migration and invasion assays

Transwell migration, Matrigel invasion, and Wound healing scratch assays were used to determine migration and invasion of the indicated cells. For the migration and Matrigel invasion assay, 1 × 10^5^ cells and 5 × 10^4^ cells were separately evaluated by Transwell Permeable Support (Corning, NY, USA). The detailed experimental protocol was based on previously published reference [65].

For the wound healing scratch assay, cells were generated a scratch after being cultured for 24 h. Next, cells were washed with PBS to remove detached cells. Images were captured on fluorescent microscopy (Nikon Eclipse Ti, Tokyo, Japan) connected to a Nikon camera using NIS-Elements software (NIS) at the respective time points (24, 48, and 72 h).

### Animal experiments

#### Animal xenograft tumor

To establish xenograft tumors, 5 × 10^6^ Neogenin-expressing or control cells (CRC HCT 116 and Glioma U87MG) were suspended at a 1:1 ratio in 100 μL of complete medium and Matrigel (BD Biosciences) and delivered via subcutaneous injections into BALB/c nude mice. The size of subcutaneous tumors was measured twice 1 week, and the volume was calculated using the standard modified formula Volume (mm^3^) = (length × height^2^) × π/6. Then, tumor formation was observed for the following 1 month.

#### Animal lung metastasis model

For tumor metastasis, nude mice were injected with 2 × 10^6^ HCT 116 cells overexpressed either EV or NEO1 were implanted into the lateral vein in the nude mouse tail (*n* = 5 mice per group). After 6 weeks, the lung tissues of mice were collected for metastatic foci evaluation and IHC study.

The 5–6-week male athymic BALB/c nude mice were purchased from the Shanghai Experimental Animal Center of the Chinese Academic of Sciences (Shanghai, P.R.C) and were maintained under defined conditions at the Animal Experiment Center of Soochow University. All animal experiments were approved by the Animal Care and Use Committee of Soochow University.

### Bioinformatic analysis

#### Shannon’s entropy and multivariate mutual information

Shannon’s entropy and multivariate mutual information were calculated as described previously [66] with modification. To calculate Shannon’s entropy in each tumor type, the values of the enriched score or gene expression in that tumor type were first normalized to the total value, and then log-transformed. Before the transformation, zero values were converted into 1. Here, we hold the assumption that the distribution is discrete, and the calculation follows:

$$S\left( X \right) = - \mathop {\sum}\limits_{n = 1}^n {P\left( {X_n} \right)Log_2\left( {P\left( {X_n} \right)} \right)}$$

The mutual information was then computed in each tumor sample or cell of that tumor type. Here we defined SA as a group of Shannon’s entropy values of the enriched score and the testing gene in the test tumor type: SA = {S(X1), S(X_2_), S (X_3_),….. S(X_n_)}, SG is all subgroups belonging to SA, SG_num_ is the number of SG, and the total item number in SA should be at least two, then we can calculate the mutual information via:

$${\mathrm{MI}} \left( {{\mathrm{SA}} } \right)^n = - \mathop {\sum }\limits_{{\mathrm{SG}} } \left( { - 1} \right)^{{\mathrm{SGnum}} }{\mathrm{SG}}$$

In this study, we calculated the mutual information of two variants and three variants. The final normalized mutual information was calculated by dividing the non-negative square root of Shannon’s entropy values of all calculated variants. The co-associated receptor genes were further enriched by comparing with the defined ligand-receptor information of each gene as described in [67].

#### The activity score analysis of the cell cycle and EMT

The activity score analysis was performed as described previously with modifications [68]. For each tumor type, the significant functional attribute (mitotic or EMT) activation was evaluated by comparing the expression of functional attribute-related genes and the expression of random genes. Briefly, the gene expression of each sample was normalized to a total of one million counts. The most variable genes of each tumor type were enriched by estimating the mean and coefficients of variation. The most variable functional attribute genes were selected for activity scoring, and the rest genes were ranked by the expression and divided into 25 intervals based on the rank. Next, we selected the first 100 genes in each interval for randomization to generate the random gene matrix. The activity scores were generated by estimating the differential mean expression of the functional attribute genes and the randomized genes. Thus, each tumor sample was assigned to the activity score of the functional attribute, and subsequently used for further calculation.

#### Correlation, pathway, and survival analysis

Co-expression analysis was performed using Spearman’s rank correlation coefficient (SciPy package) between the expression of Neogenin and all other genes in the dimension of all tested samples. The correlated genes were selected upon the criteria that a significant correlation existed in at least two out of eight datasets. The single-cell RNA sequencing data was extracted from [68]. TCGA pan-cancer data was extracted from https://gdc.cancer.gov/about-data/publications/ pancanatlas. Pathway enrichment was assessed using Python package jdrudolph/goenrich and PyPathway with modification. The Lifelines package was used for Kaplan–Meier survival analysis and generated by Matplotlib and Seaborn packages.

### Statistical analysis

All statistical values were measured using SPSS 22.0 software (Chicago, IL, USA) or GraphPad Prism 7.0. For comparison, the *T*-test (for parametric data) and the Mann–Whitney *U*-test (for nonparametric data) were used to determine statistical significance. In instances where multiple comparisons were performed, the one-way ANOVA test (for parametric data) or Kruskal–Wallis test (for nonparametric data) was used. In addition, the Spearman rank correlation test and Kaplan–Meier method were separately employed for correlation analyses and survival curves. Results are reported as mean ± standard deviation (SD). All IHC, IP, western blot, and cellular function assays were conducted ≥3 times to ensure reproducibility. *P* values <0.05 were considered statistically significant, with *p < 0.05, **p < 0.001, and ***p < 0.0001, unless otherwise indicated in the figures.

## Supplementary information

Supplementary Figure legends

Supplementary Fig.S1

Supplementary Fig.S2

Supplementary Fig.S3

Supplementary Fig.S4

Supplementary Fig.S5

Supplementary Fig.S6

Supplementary Fig.S7

Supplementary Table S1

Supplementary Table S2

Supplementary Table S3

Supplementary Table S4

Supplementary Table S5

Supplementary Table S6

Original Data File

## Data Availability

The datasets used and/or analyzed during the current study are available from the corresponding author upon reasonable request. Original western blots are attached as Supplemental materials.
