# Supplementary material for: Neogenin suppresses tumor progression and metastasis via inhibiting Merlin/YAP signaling
Source: Cell Death Discov. 2023 Feb 6;9:47. doi: 10.1038/s41420-023-01345-w (PMC9902585; doi:10.1038/s41420-023-01345-w)
Supplement: Supplementary file 1 — Supplementary Figure legends [file 41420_2023_1345_MOESM1_ESM.docx]

**Supplementary Figure legends**

**Supplementary Fig. S1 The expression of Neogenin in CRC and Glioma. A** IHC analysis of Neogenin expression in Glioma tissues. **B** Immunoblotting analysis of Neogenin expression in six CRC and two Glioma cell lines. The GAPDH was used as a loading control.

**Supplementary Fig. S2 Neogenin affects downstream signaling related to EMT progress. A** Western blotting (WB) analysis of Neogenin protein level in total cell lysates of HCT 116, RKO, U251, and U87 MG cells with either vector or NEO1 stably expressed. **B, C** Western blotting (WB) analysis of Neogenin and Vimentin protein level in control versus NEO1-expressing HCT 116 (B) or U87 MG (C) cells.

**Supplementary Fig. S3 Neogenin affects the EMT progress of CRC SW480 cells. A** Quantitative RT-PCR for EMT related genes and NEO1 in SW480 cells transduced with siRNA for NEO1(si1816, si2920). siNC was used as control. **B** The SW480 cells transduced with siRNA for NEO1 were applied for migration assay. Scale bars, 200 μm. Data represent means ± SD. **p* < 0.05, ***p* < 0.001compared with siNC group by Multiple t-tests (A) or two-tailed Student’s t-test (B).

**Supplementary Fig. S4 Neogenin Suppresses the malignant behaviors of Glioma cells. A-D** NEO1 suppresses the motility of Glioma cells. U87 MG and U251 cells transduced with Lentivirus for NEO1 were applied for transwell assay (A, B) and scratch assay (C, D). Bar chat represents the area quantification of migrated cells. Scale bars, 100 μm, 500 μm. **E** NEO1 suppresses the EMT of U87 MG and U251. Quantitative RT-PCR for EMT related genes and NEO1 in U87 MG and U251 cells transduced with Lentivirus. EV was used as control. **F** Subcutaneous growth of control and NEO1-expressing U87 MG cells in Nude mice. 5×10^6^ cells per injection, n=3 mice per group. The primary tumor growth rate in different experimental groups in measured once per week. Experiments were performed in triplicate. **G** Representative images of tumors from Fig. S3F. **H** Primary tumor samples from the mice in Fig. S3F were collected for IHC staining of Ki67. n = 3 per group. Scale bars, 50 μm. Data represent means ± SD. **p* < 0.05, ***p* < 0.001, ****p* < 0.0001. Significance determined by two-tailed Student’s t-test (A-E).

**Supplementary Fig. S5** **Alpha Fold Multimer prediction between Neogenin and Merlin. A** Visualization in structure. The structure of Merlin was shown in green, while the intracellular domain of Neogenin was shown in pink. The red cycle showed the connection between Neogenin and Merlin. **B** The secondary structure visualization. Beta-sheets and helices were colored.

**Supplementary Fig. S6** **Neogenin/Merlin impedes the EMT progress in Glioma. A** Representative image of IHC staining (up) and the correlation between Neogenin and Merlin (down) in primary tumors from Glioma patients. Scale bars, 50 μm. **B** NEO1 suppresses the EMT of U87 MG cells, but interfering with NF2 expression reverses the EMT. Quantitative RT-PCR for EMT-related genes and NEO1 in U87 MG cells transduced with Lentivirus for NEO1 and siRNA for NF2. EV and siNC were used as control. **C** NEO1 suppresses the migration of Glioma cells, but interfering with merlin expression reverses the inhibitory effect. Glioma U251 cells transduced with Lentivirus for NEO1 and siRNA for Merlin were applied for assay. siNC was used as control. Scale bars, 100 μm. **D** NEO1 suppresses the motility of Glioma cells, but interfering with NF2 expression reverses the inhibitory effect. U251 cells transduced with Lentivirus for NEO1 and siRNA for NF2 were applied for scratch assay. siNC was used as control. Scale bars, 500 μm. **E** NEO1 suppresses the invasion of Glioma cells, but interfering with merlin expression reverses the inhibitory effect. Glioma U87 MG cells transduced with Lentivirus for NEO1 and siRNA for NF2 were applied for invasion assay. siNC was used as control. Scale bars, 500 μm. Data represent means ± SD. **p* < 0.05, ***p* < 0.001, ****p* < 0.0001 compared with EV group by Multiple t-tests (B) or two-tailed Student’s t-test (C-E). ^#^*p* < 0.05, ^##^*p* < 0.001, ^###^*p* < 0.0001 compared with NEO1/siNC group.

**Supplementary Fig. S7 Neogenin exerts its Tumor-Suppressive function by Merlin/YAP Signaling Pathways in Glioma. A** Western blot of p-YAP, YAP, Neogenin, merlin, and GAPDH in Glioma U251 and U87 MG cells. NEO1 represents the overexpression of NEO1 in Glioma cells, EV as control. **B** The subcellular fractionation analysis of YAP expression in U251 cells stably expressing NEO1 (NEO1) or control (EV). Immunoblotting of GAPDH and Lamin B1 served as controls for the purity of cytoplasmic (C) and nuclear (N) fractions, respectively. **C** Representative images of IHC staining of Neogenin and YAP in tumors from transplanted Glioma model. Scale bars, 50 μm. **D** NEO1 promotes the phosphorylation of YAP in Glioma cells, but interfering with NF2 expression reverses the activated effect. U251 cells transduced with Lentivirus for NEO1 and siRNA for NF2 were applied for WB assay. siNC was used as control.
