# Supplementary figures and images for: Neogenin suppresses tumor progression and metastasis via inhibiting Merlin/YAP signaling
Source: Cell Death Discov. 2023 Feb 6;9:47. doi: 10.1038/s41420-023-01345-w (PMC9902585; doi:10.1038/s41420-023-01345-w)

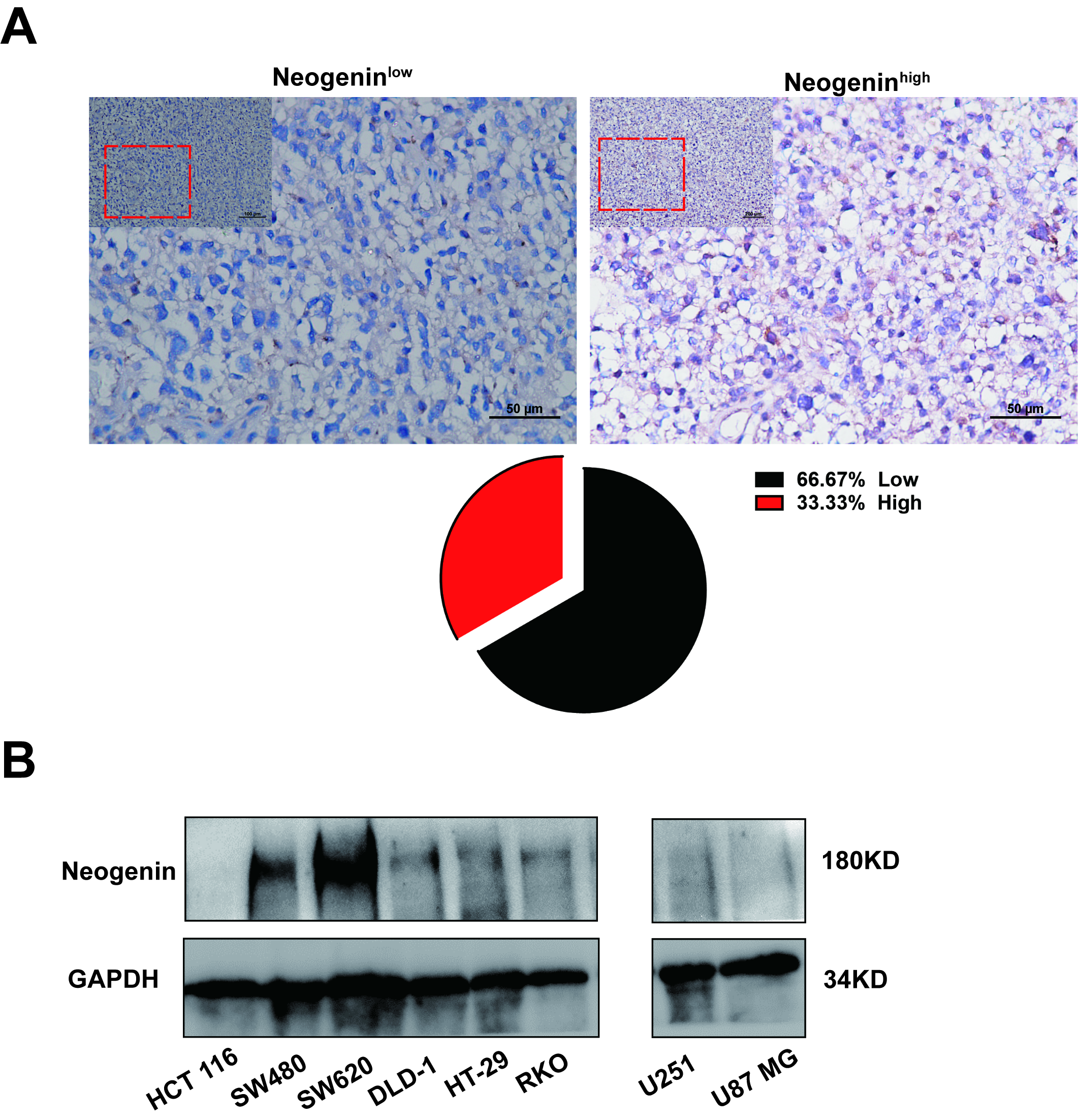

Supplement: Supplementary file 2 — Supplementary Fig.S1 [file 41420_2023_1345_MOESM2_ESM.tif]

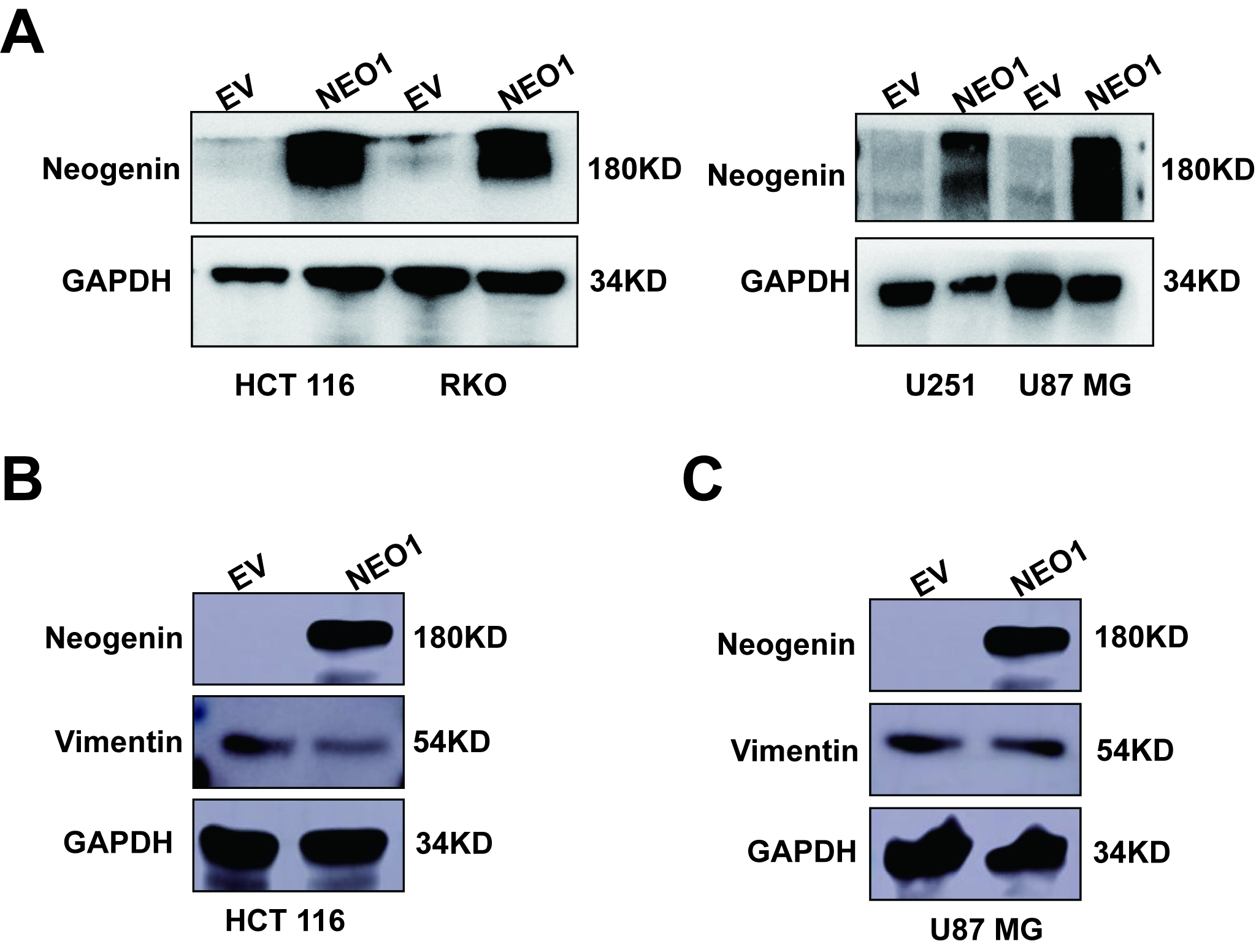

Supplement: Supplementary file 3 — Supplementary Fig.S2 [file 41420_2023_1345_MOESM3_ESM.tif]

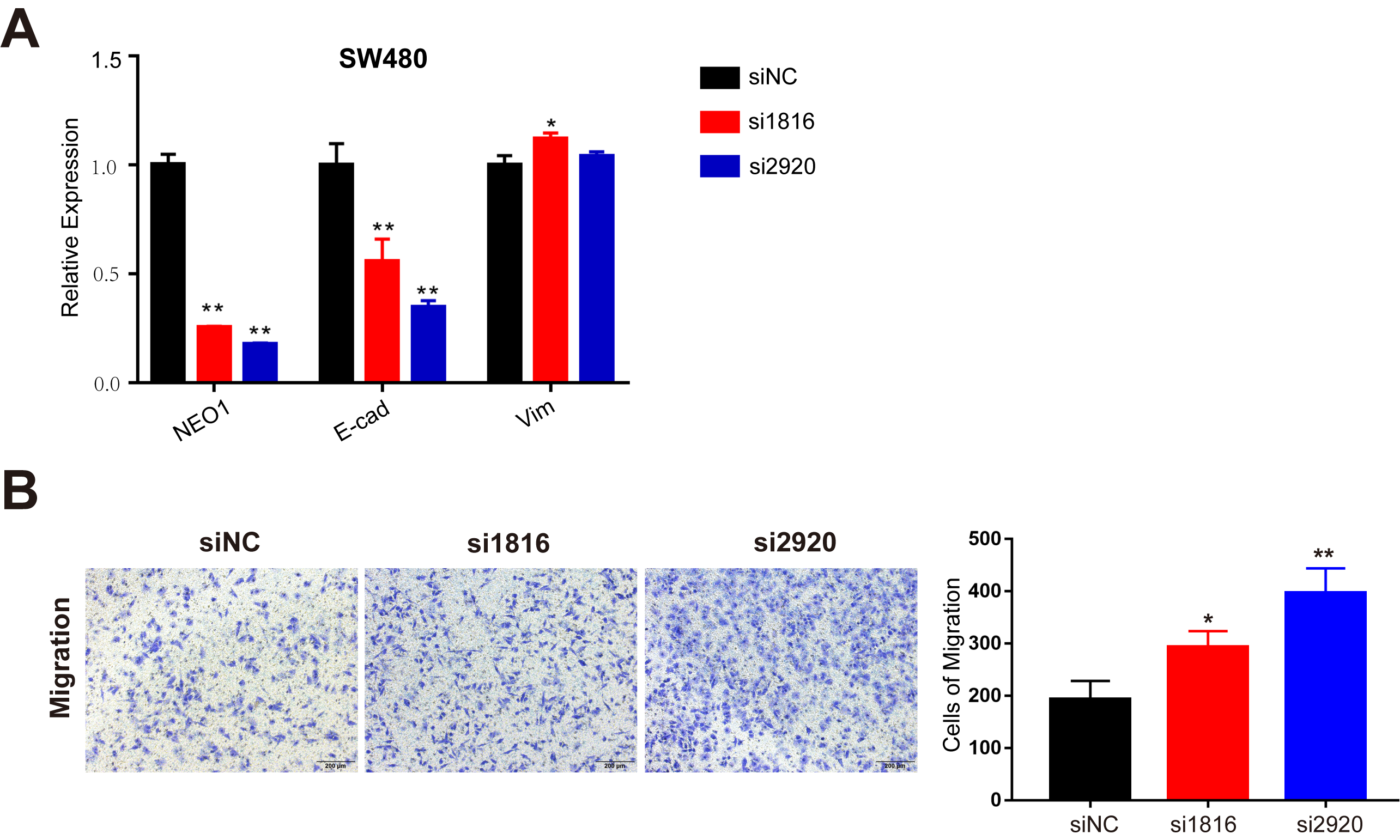

Supplement: Supplementary file 4 — Supplementary Fig.S3 [file 41420_2023_1345_MOESM4_ESM.tif]

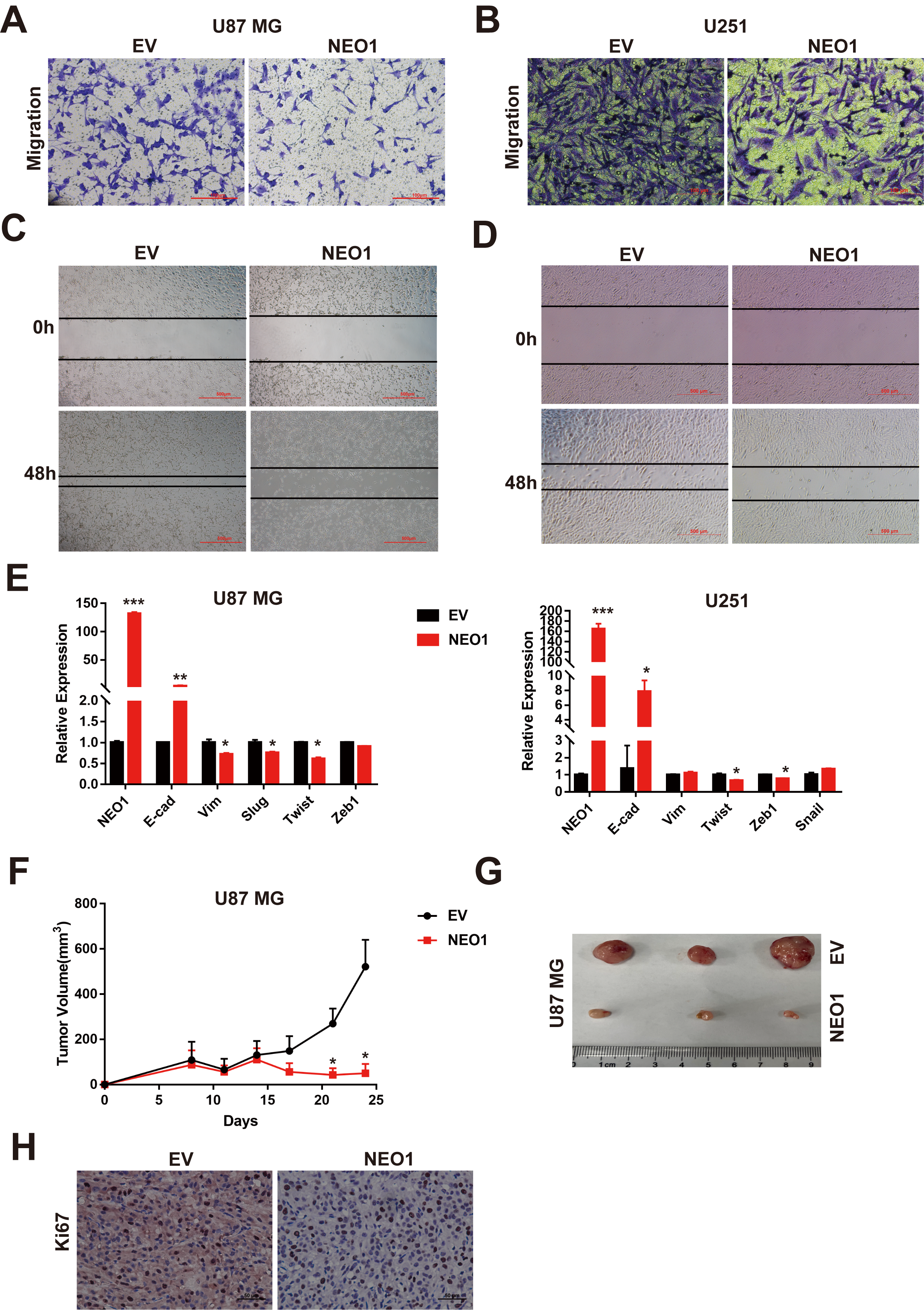

Supplement: Supplementary file 5 — Supplementary Fig.S4 [file 41420_2023_1345_MOESM5_ESM.tif]

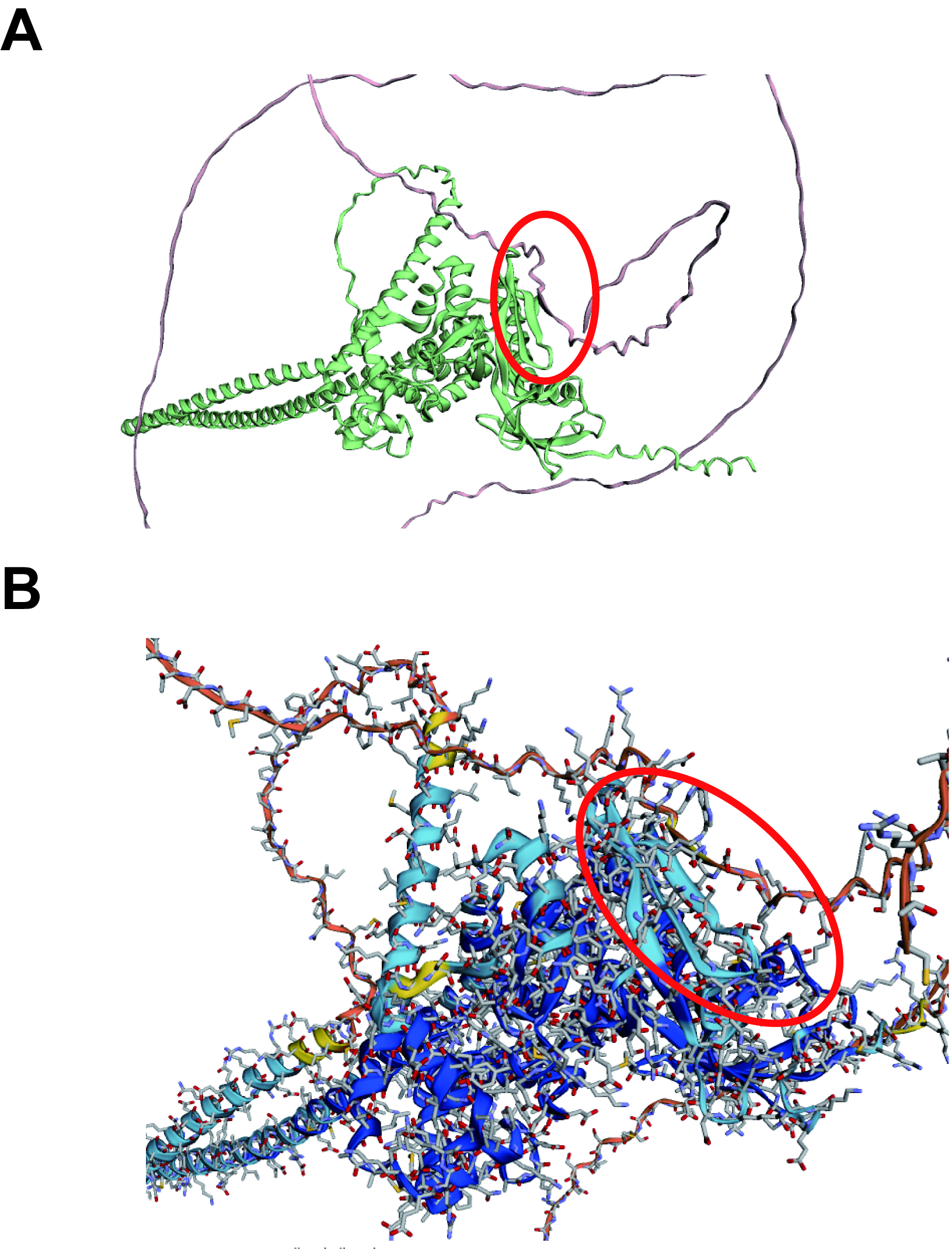

Supplement: Supplementary file 6 — Supplementary Fig.S5 [file 41420_2023_1345_MOESM6_ESM.tif]

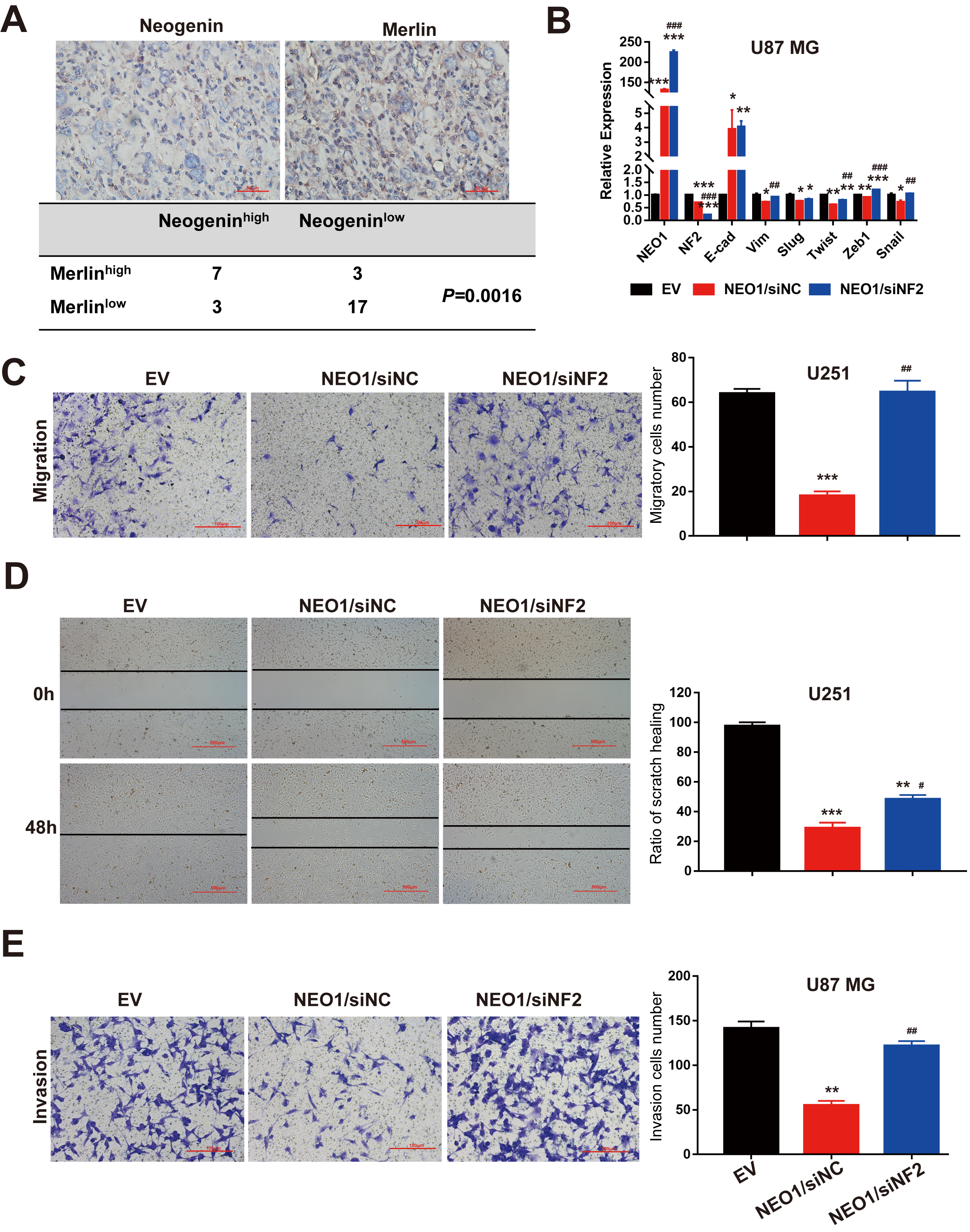

Supplement: Supplementary file 7 — Supplementary Fig.S6 [file 41420_2023_1345_MOESM7_ESM.tif]

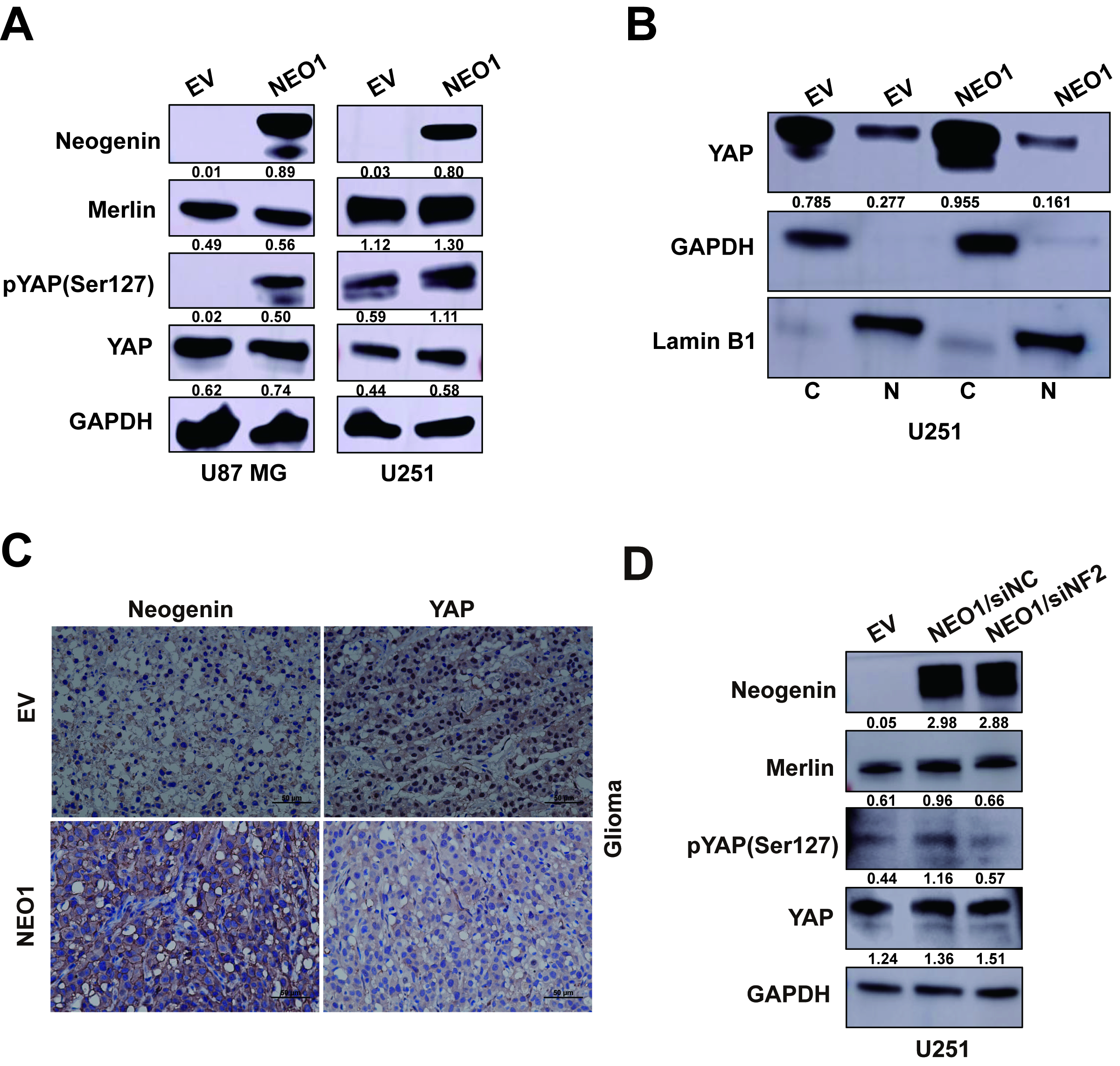

Supplement: Supplementary file 8 — Supplementary Fig.S7 [file 41420_2023_1345_MOESM8_ESM.tif]
