# Supplementary material for: Neogenin suppresses tumor progression and metastasis via inhibiting Merlin/YAP signaling
Source: Cell Death Discov. 2023 Feb 6;9:47. doi: 10.1038/s41420-023-01345-w (PMC9902585; doi:10.1038/s41420-023-01345-w)
Supplement: Supplementary file 9 — Supplementary Table S1 [file 41420_2023_1345_MOESM9_ESM.docx]

**Supplementary Tables**

**Supplementary Table S1.** Neogenin expression in correlation with CRC patients’ characteristics

|  |  | **Neogenin expression** | | |
| --- | --- | --- | --- | --- |
| **Characteristics** | **All** | **Low** | **High** | ***P* value** |
| **Gender** |  |  |  | 0.894 |
| Male | 89 | 47 | 42 |  |
| Female | 78 | 42 | 36 |  |
| **Age(years)** |  |  |  | 0.889 |
| ＜65 | 25 | 13 | 12 |  |
| ≥65 | 142 | 76 | 66 |  |
| **Tumor Volume** |  |  |  | 0.875 |
| ＜5cm^3^ | 84 | 56 | 28 |  |
| ≥5cm^3^ | 83 | 33 | 50 |  |
| **Tumor stage** |  |  |  | 0.010 |
| Ⅰ-Ⅱ | 28 | 11 | 17 |  |
| Ⅲ-Ⅳ | 139 | 78 | 61 |  |
| **LN metastases** |  |  |  | 0.010 |
| 0 | 88 | 42 | 46 |  |
| 1～3 | 38 | 17 | 21 |  |
| 4～7 | 38 | 27 | 11 |  |
| ≥7 | 3 | 3 | 0 |  |
| **Distant metastases** |  |  |  | 0.561 |
| No | 145 | 76 | 69 |  |
| Yes | 22 | 13 | 9 |  |
| **Survival** |  |  |  | 0.029 |
| Live | 70 | 34 | 36 |  |
| Dead | 97 | 55 | 42 |  |
