# Supplementary material for: Neogenin suppresses tumor progression and metastasis via inhibiting Merlin/YAP signaling
Source: Cell Death Discov. 2023 Feb 6;9:47. doi: 10.1038/s41420-023-01345-w (PMC9902585; doi:10.1038/s41420-023-01345-w)
Supplement: Supplementary file 10 — Supplementary Table S2 [file 41420_2023_1345_MOESM10_ESM.docx]

**Supplementary Tables**

**Supplementary Table S2.** Sequences of siRNAs

| **Sequences** |
| --- |
| **siRNA sense(5’→3’) antisense（5’→3’）** |
| siNC UUCUCCGAACGUGUCACGUTT ACGUGACACGUUCGGAGAATT |
| si1246 GCUCACGAAUCUAUGGAUATT UAUCCAUAGAUUCGUGAGCTT |
| si1816 GCACCUAACCUUCGUGCAUTT AUGCACGAAGGUUAGGUGCTT |
| si2920 GCAACCACUUUGAGUUAUUTT AAUAACUCAAAGUGGUUGCTT |
| si774 GGAGAUCACACAACAUUUATT UAAAUGUUGUGUGAUCUCCTT |
| si628 GGACUGCAGUACACAAUCATT UGAUUGUGUACUGCAGUCCTT |
| si1952 GCCUGUCUUUCGACUUCAATT UUGAAGUCGAAAGACAGGCTT |

**Abbreviations:** The Small interfering RNA (siRNA) targeting specific human NEO1 sites (si1246, si1816 and si2920) and negative control siRNA (siNC) were synthesized by GenePharma (Shanghai, China). Similarly, the siRNA targeting specific human NF2 sites were si774, si628 and si1952.
