# Supplementary material for: Neogenin suppresses tumor progression and metastasis via inhibiting Merlin/YAP signaling
Source: Cell Death Discov. 2023 Feb 6;9:47. doi: 10.1038/s41420-023-01345-w (PMC9902585; doi:10.1038/s41420-023-01345-w)
Supplement: Supplementary file 11 — Supplementary Table S3 [file 41420_2023_1345_MOESM11_ESM.docx]

**Supplementary Tables**

**Supplementary Table S3.** Gene Expression (Quantitative RT-PCR) primers

| **Gene** | **Forward primer (5’- 3’)** | **Reverse primer (5’- 3’)** |
| --- | --- | --- |
| NEO1 | GGAGCCGGTGGATACACTCT | TGGCGTCGATCATCTGATACTA |
| NF2 | TTGCGAGATGAAGTGGAAAGG | CAAGAAGTGAAAGGTGACTGGTT |
| Actin | CATGTACGTTGCTATCCAGGC | CTCCTTAATGTCACGCACGAT |
| E-cad | CGAGAGCTACACGTTCACGG | GGGTGTCGAGGGAAAAATAGG |
| Vim | AGTCCACTGAGTACCGGAGAC | CATTTCACGCATCTGGCGTTC |
| Snail | TCGGAAGCCTAACTACAGCGA | AGATGAGCATTGGCAGCGAG |
| Slug | TGTGACAAGGAATATGTGAGCC | TGAGCCCTCAGATTTGACCTG |
| Twist | GTCCGCAGTCTTACGAGGAG | GCTTGAGGGTCTGAATCTTGCT |
| Zeb1 | CAGCTTGATACCTGTGAATGGG | TATCTGTGGTCGTGTGGGACT |
