# Supplementary material for: Neogenin suppresses tumor progression and metastasis via inhibiting Merlin/YAP signaling
Source: Cell Death Discov. 2023 Feb 6;9:47. doi: 10.1038/s41420-023-01345-w (PMC9902585; doi:10.1038/s41420-023-01345-w)
Supplement: Supplementary file 13 — Supplementary Table S5 [file 41420_2023_1345_MOESM13_ESM.docx]

**Supplementary Tables**

**Supplementary Table S5.** The mRNA transcriptome sequencing data of 8 tumor models from TCGA and other databases

| **Cancer type** | **Case**  **number** | **%**  **Positive** | **Correlation** | ***P* value**  **Overall Survival** |
| --- | --- | --- | --- | --- |
| **Colon cancer** | 285 | 62.4 | Negative | 0.012 |
| **Glioma** | 273 | 3.66 | Negative | ＜0.0001 |
| **Lung cancer** | 106 | 76.4 | Negative | 0.014 |
| **Kidney Renal Clear Cell Carcinoma** | 533 | 68.7 | Negative | ＜0.0001 |
| **Neuroblastoma** | 498 | 50 | Negative | ＜0.0001 |
| **Liver Hepatocellular Carcinoma** | 371 | 77.6 | Negative | ＜0.0001 |
| **Breast cancer** | 104 | 77.9 | Negative | 0.048 |
| **Melanoma** | 214 | 86.9 | Negative | 0.038 |
