# Supplementary material for: Neogenin suppresses tumor progression and metastasis via inhibiting Merlin/YAP signaling
Source: Cell Death Discov. 2023 Feb 6;9:47. doi: 10.1038/s41420-023-01345-w (PMC9902585; doi:10.1038/s41420-023-01345-w)
Supplement: Supplementary file 15 — Original Data File [file 41420_2023_1345_MOESM15_ESM.pdf]

Fig.4C

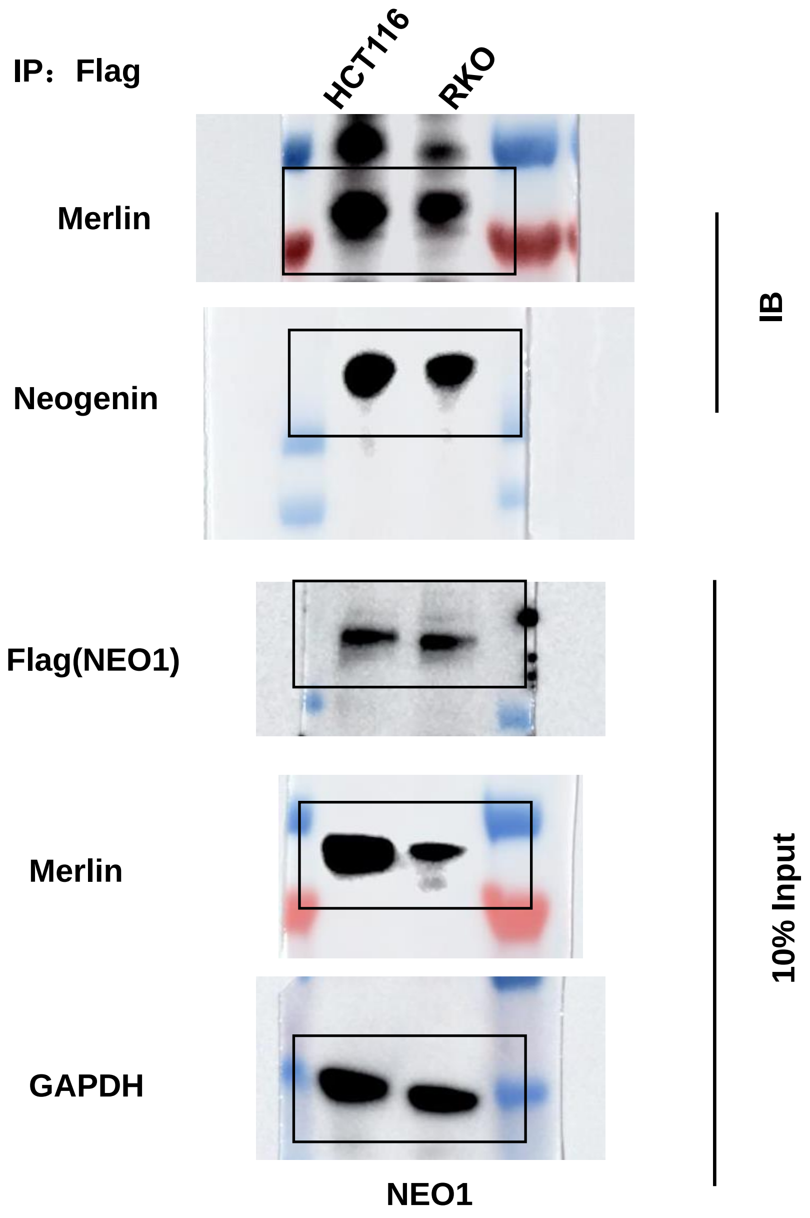
IP：Flag
HCT116
RKO
Merlin
Neogenin
IB
Flag(NEO1)
Merlin
GAPDH
10% Input
NEO1

Fig.4D

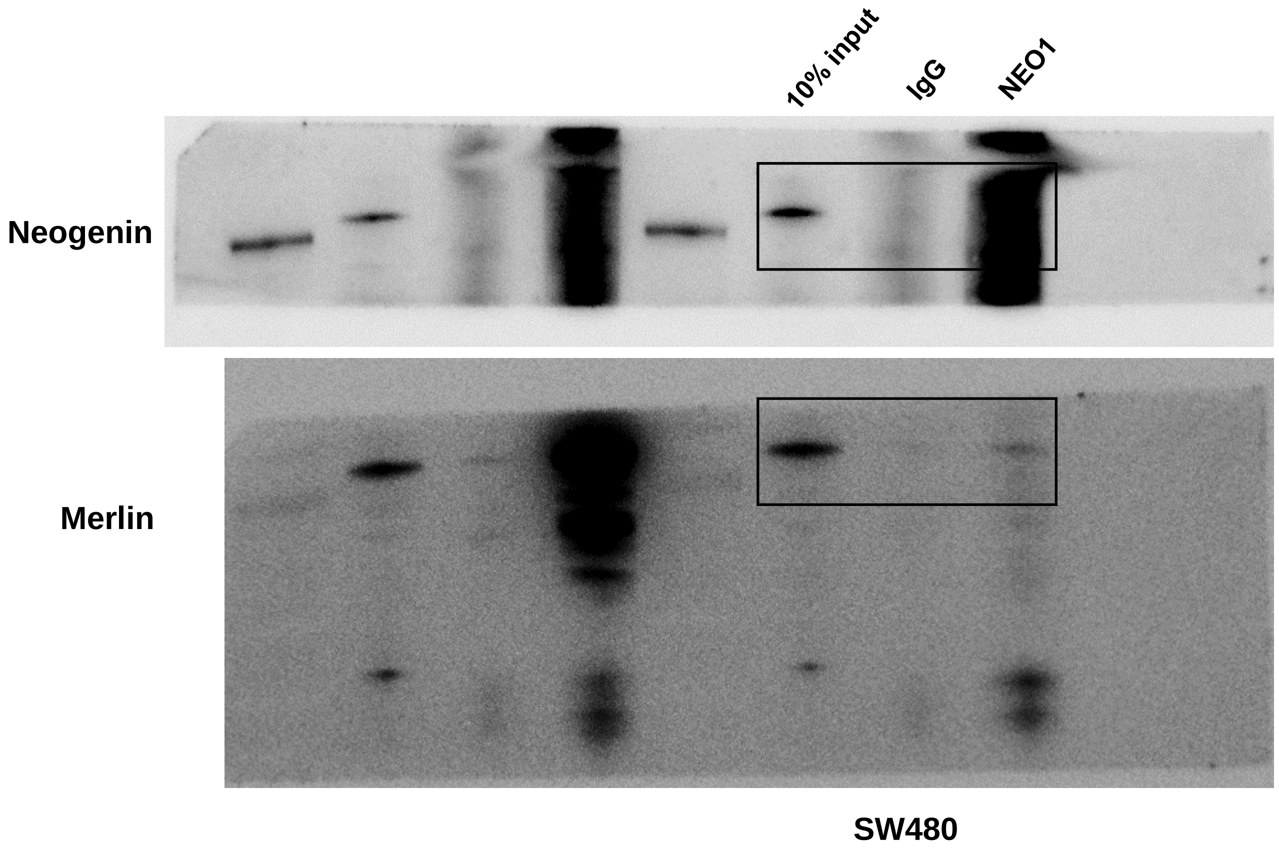

Fig.5B

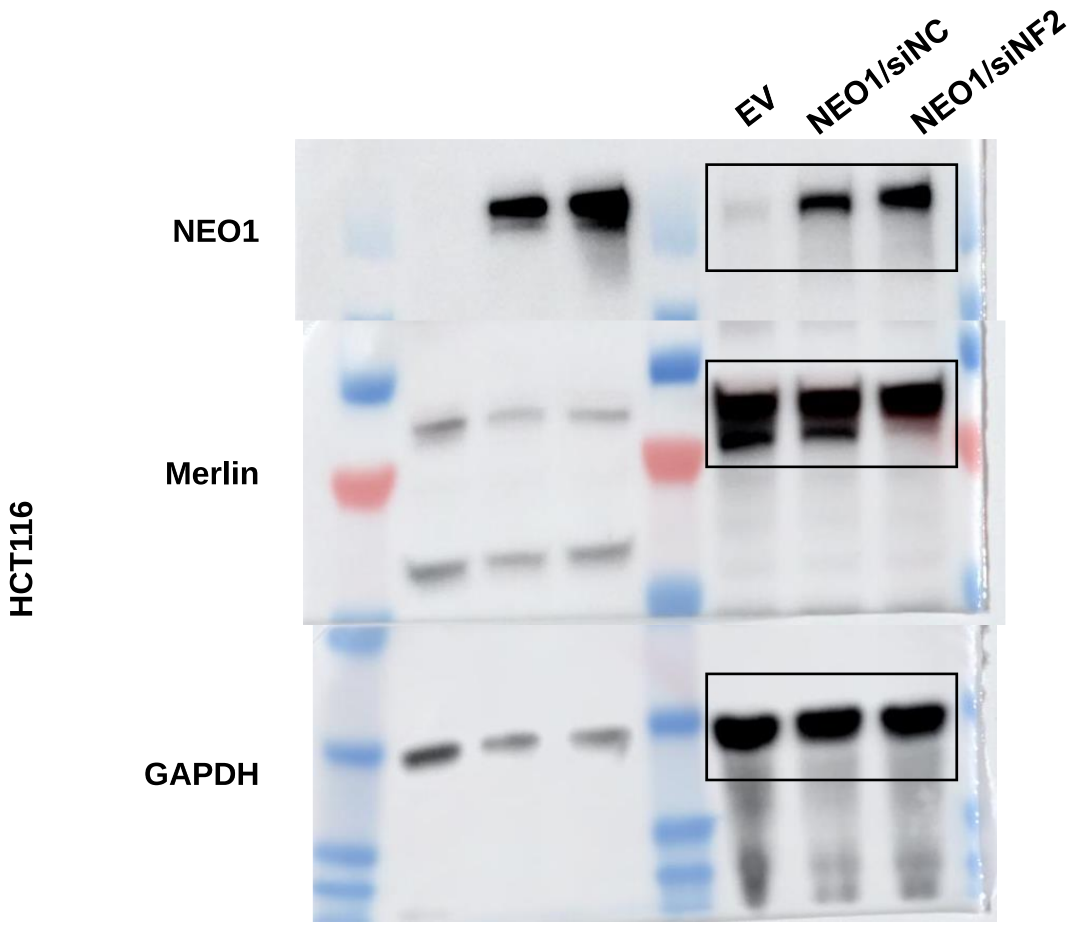
EV
NEO1/siNC
NEO1/siNF2
NEO1
Merlin
GAPDH
HCT116

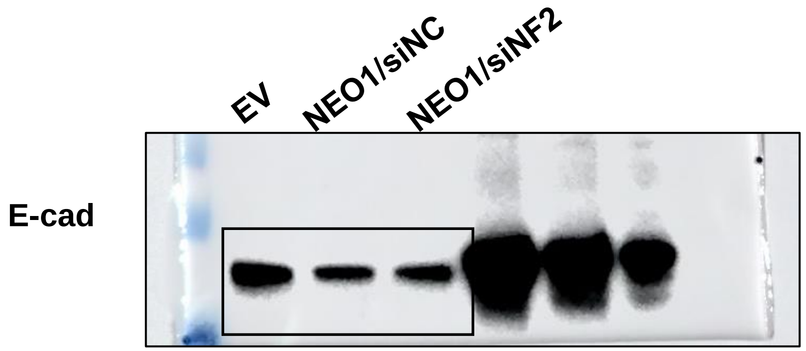
EV
NEO1/siNC
NEO1/siNF2
E-cad

Fig.6B

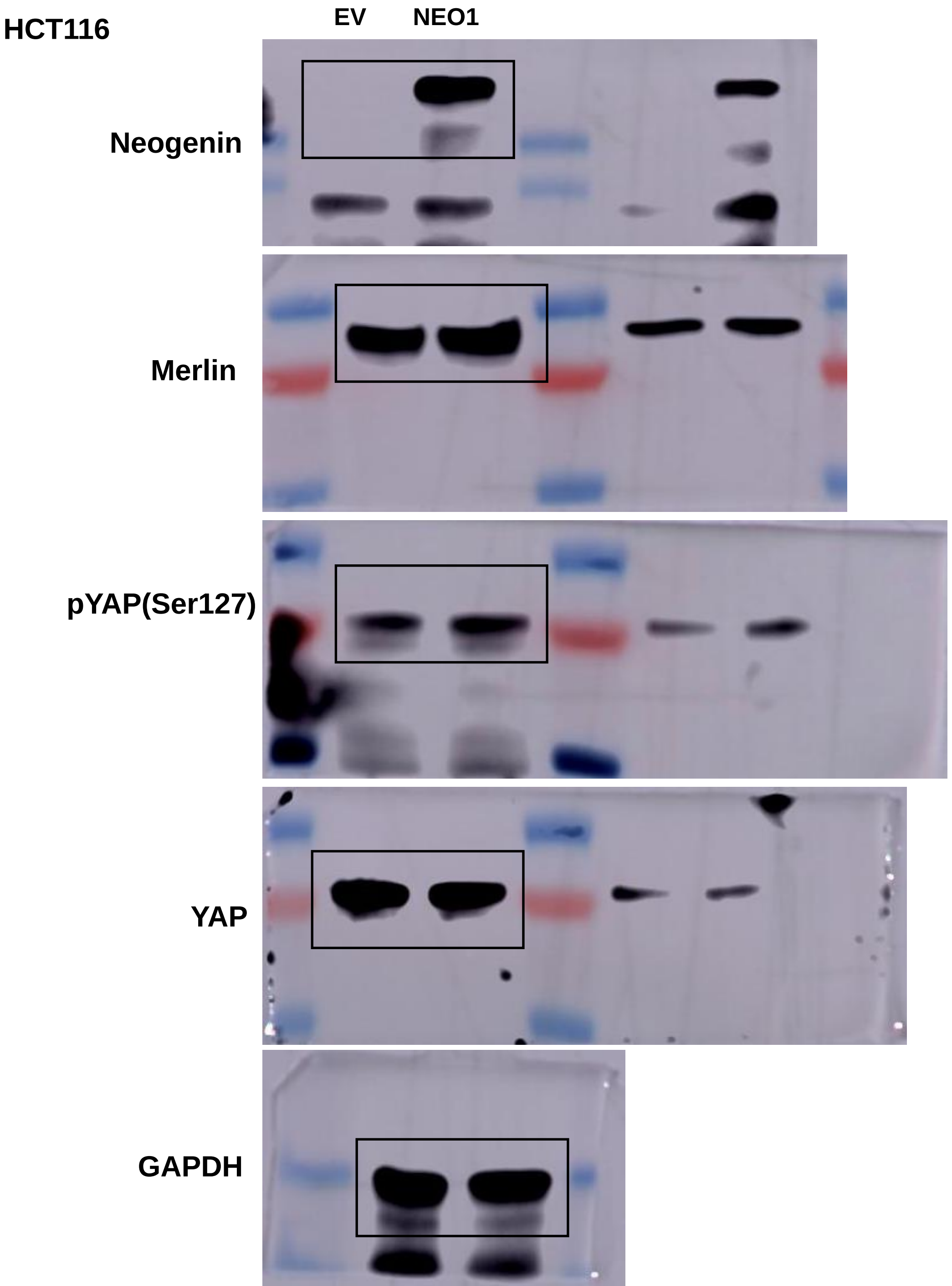

Fig.6B

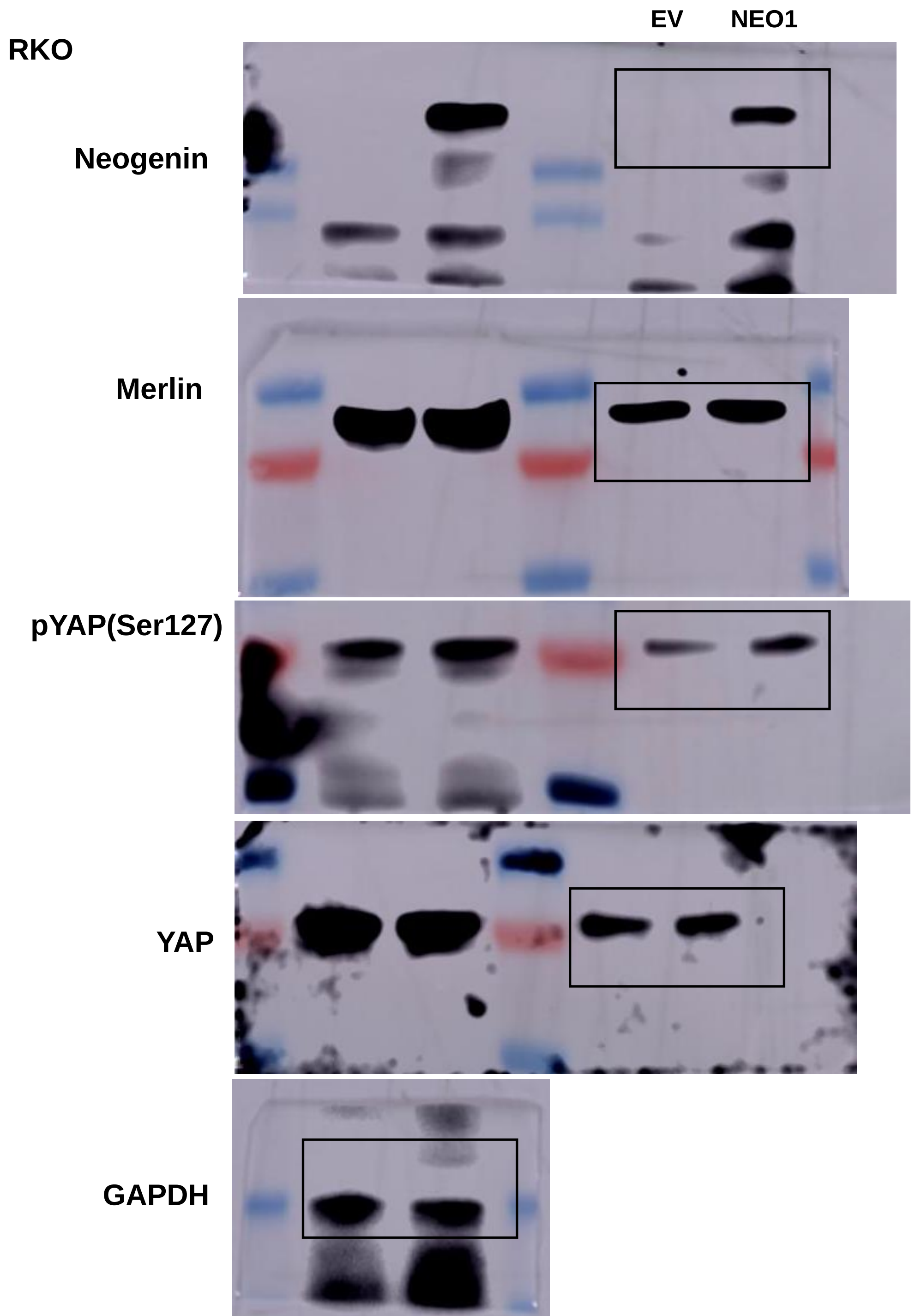

Fig.6C

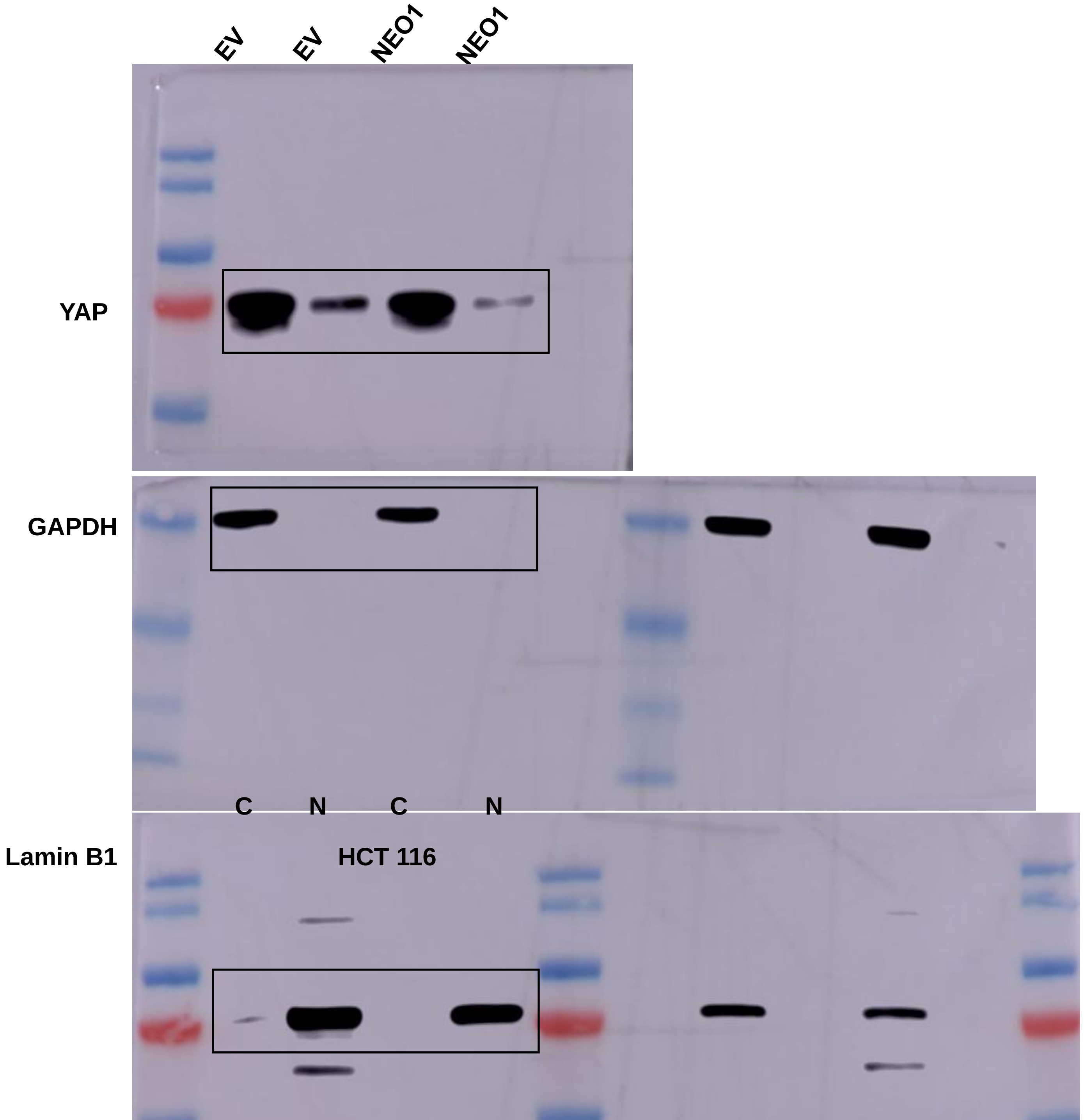

Fig.6D

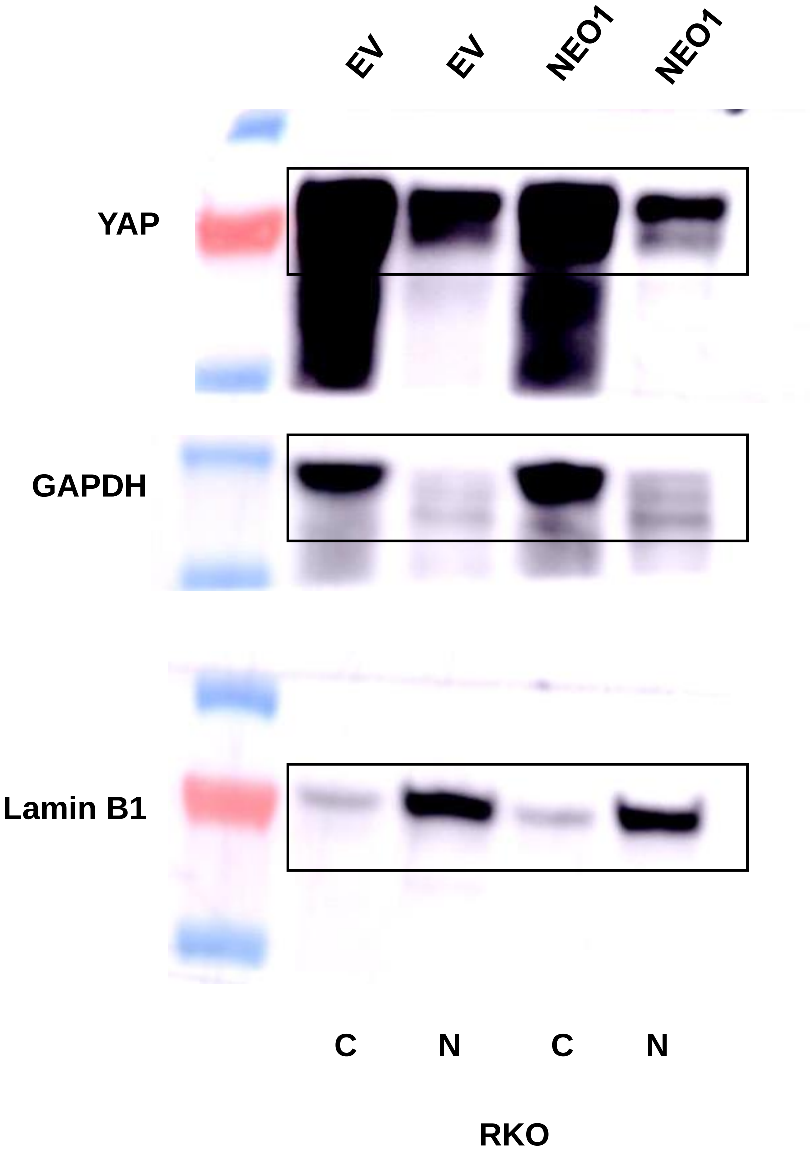

Fig.6G

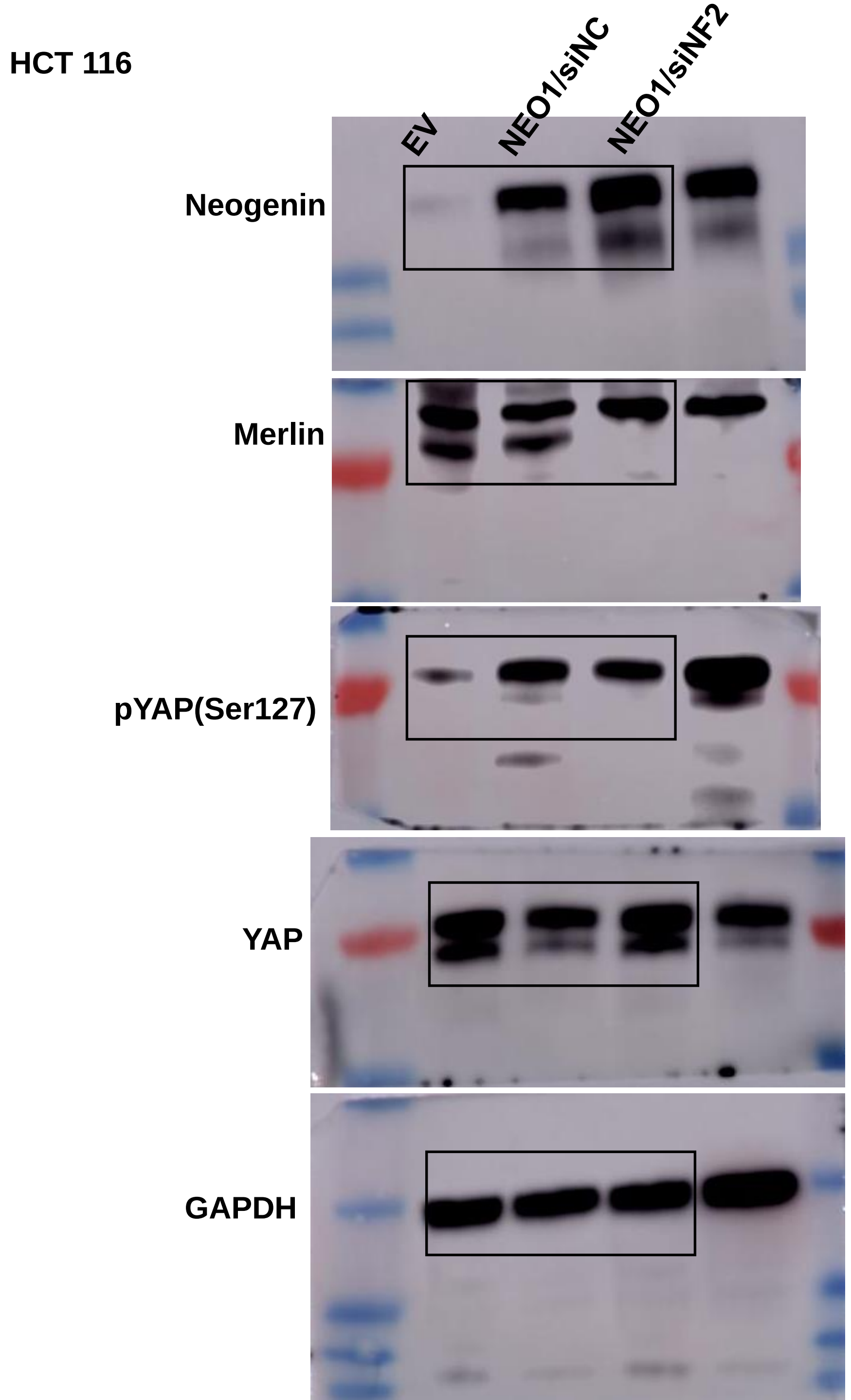
HCT 116
EV
NEO1/siNC
NEO1/siNF2
Neogenin
Merlin
pYAP(Ser127)
YAP
GAPDH

Fig.6G

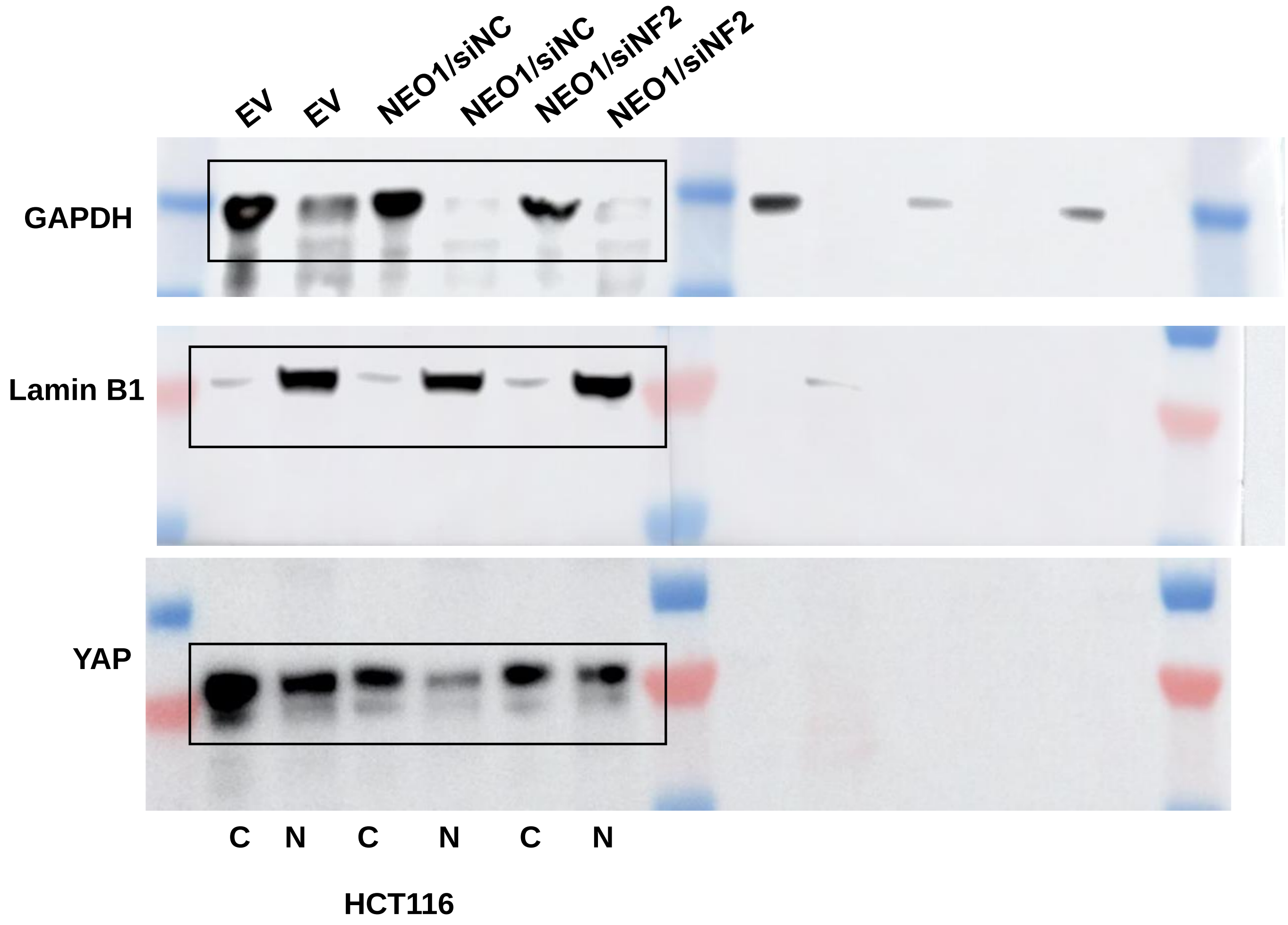
EV
EV
NEO1/siNC
NEO1/siNC
NEO1/siNF2
NEO1/siNF2
GAPDH
Lamin B1
YAP
C
N
C
N
C
N
HCT116

Fig.S1B

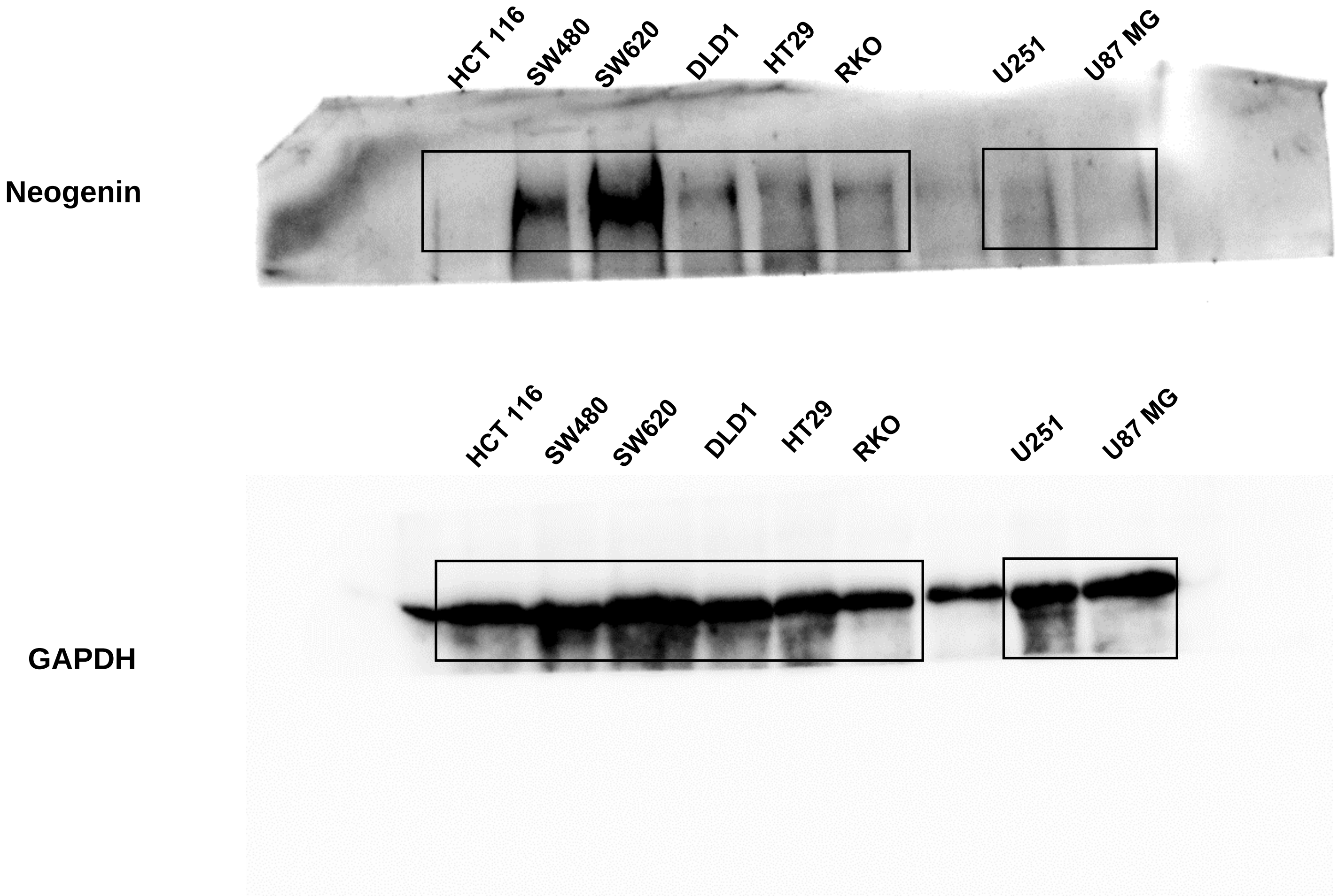

HCT 116
SW480
SW620
DLD1
HT29
RKO
U251
U87 MG
Neogenin
HCT 116
SW480
SW620
DLD1
HT29
RKO
U251
U87 MG
GAPDH

Fig.S2A

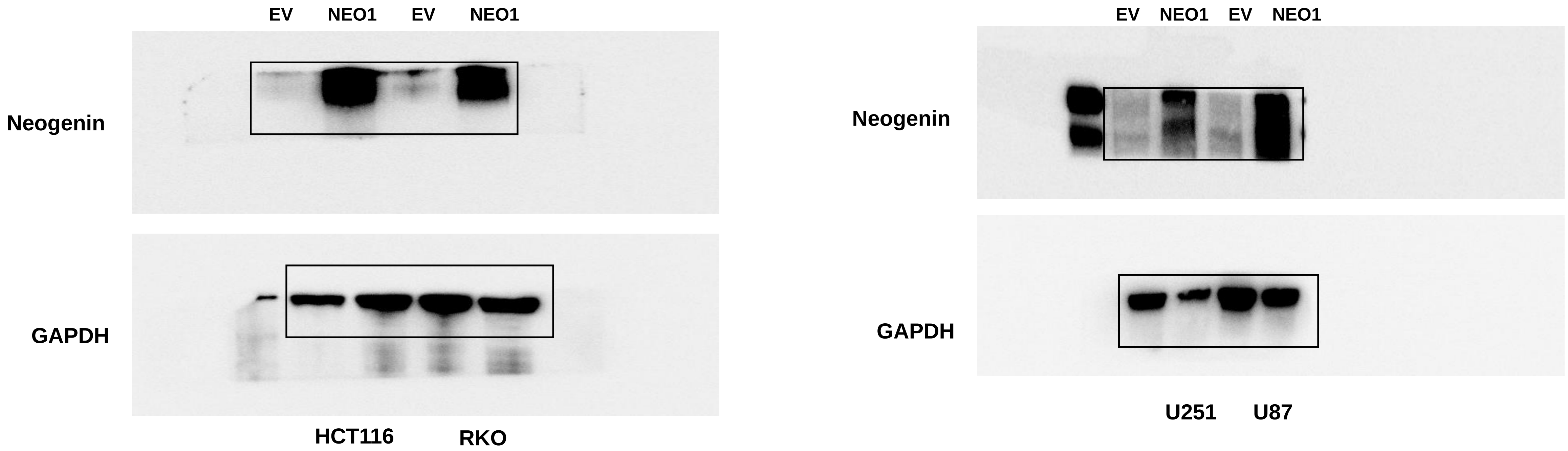
EV
NEO1
EV
NEO1
Neogenin
GAPDH
HCT116
RKO
EV
NEO1
EV
NEO1
Neogenin
GAPDH
U251
U87

Fig.S2B

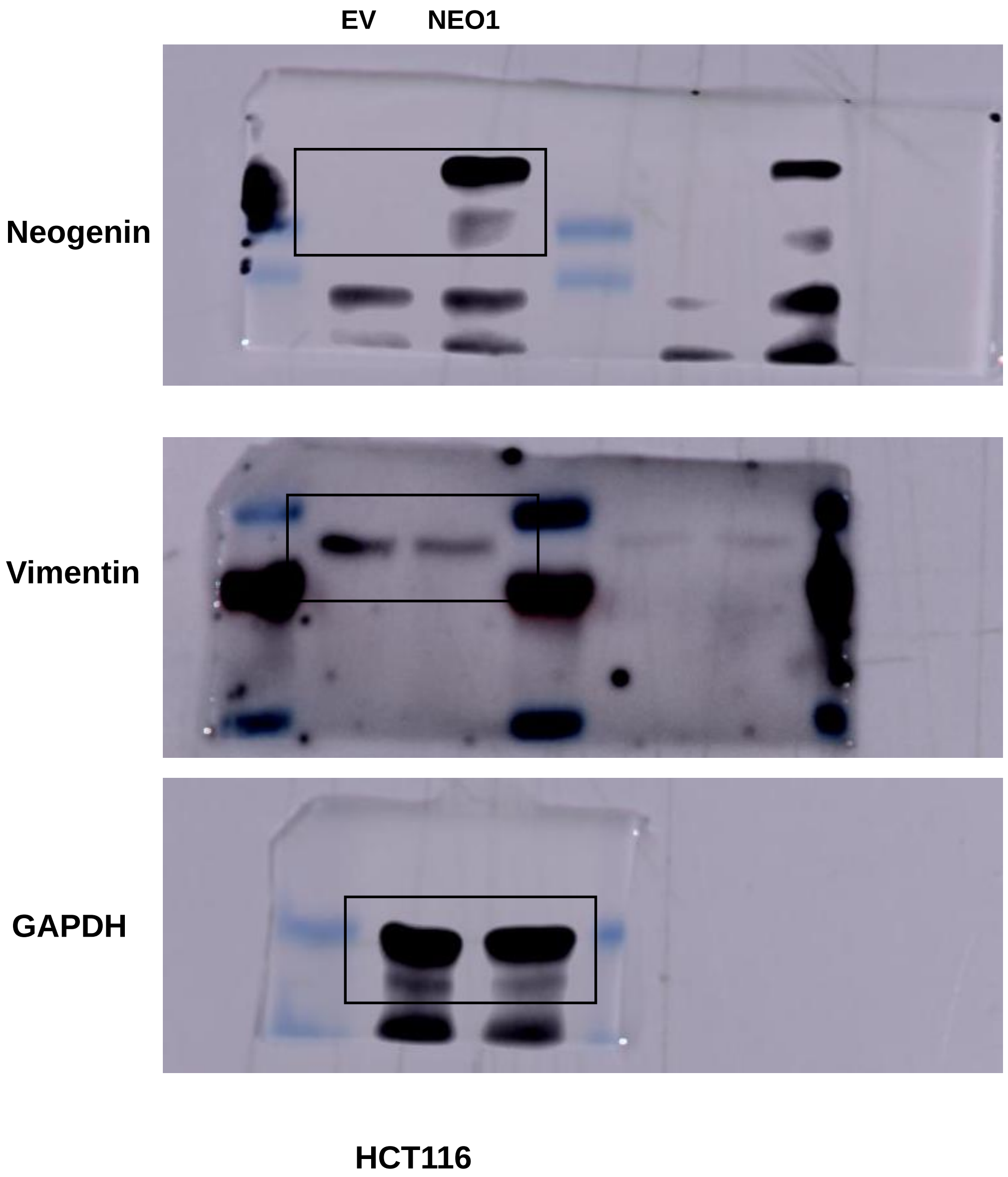
EV
NEO1
Neogenin
Vimentin
GAPDH
HCT116

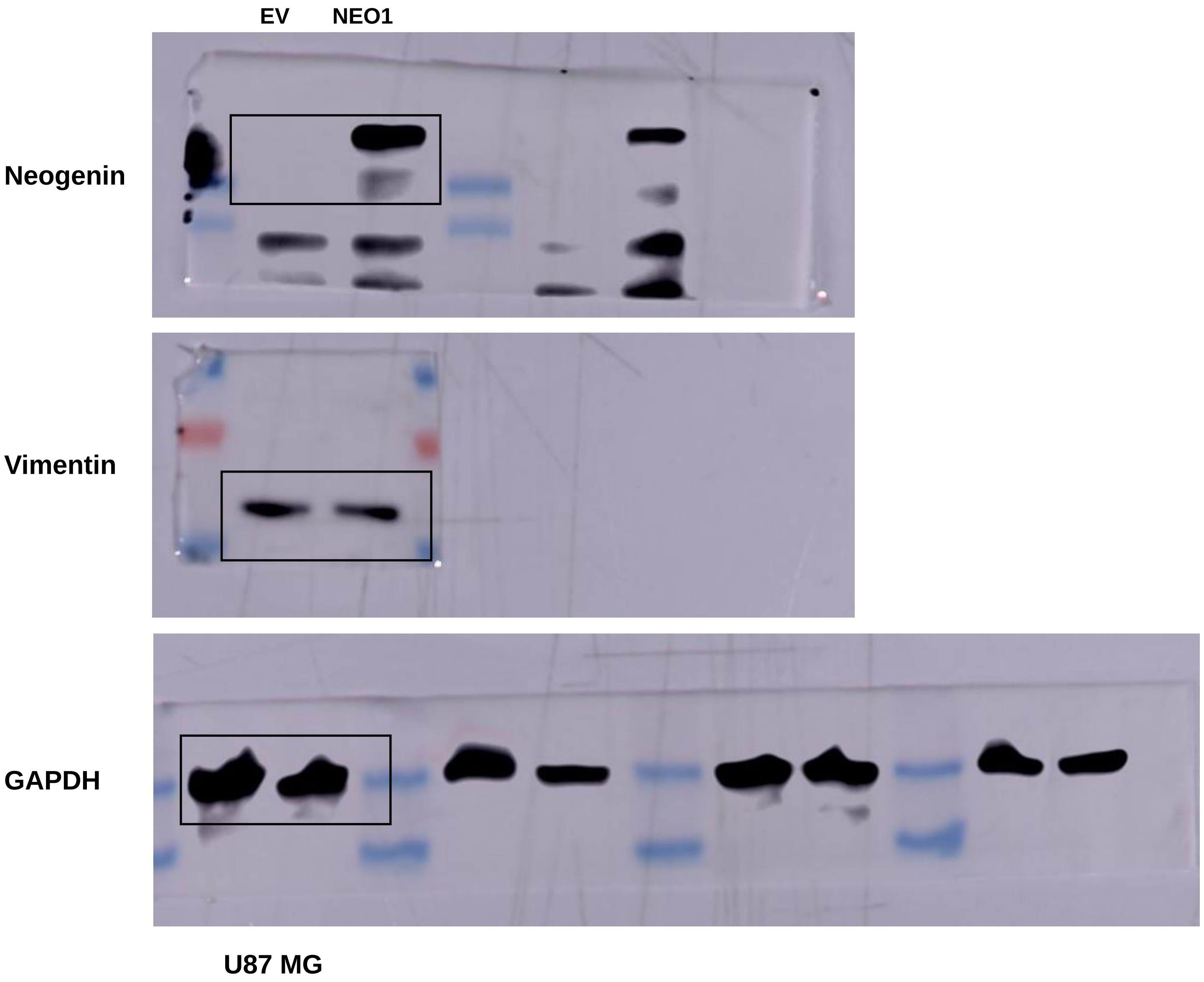
EV
NEO1
Neogenin
Vimentin
GAPDH
U87 MG

Fig.S7A

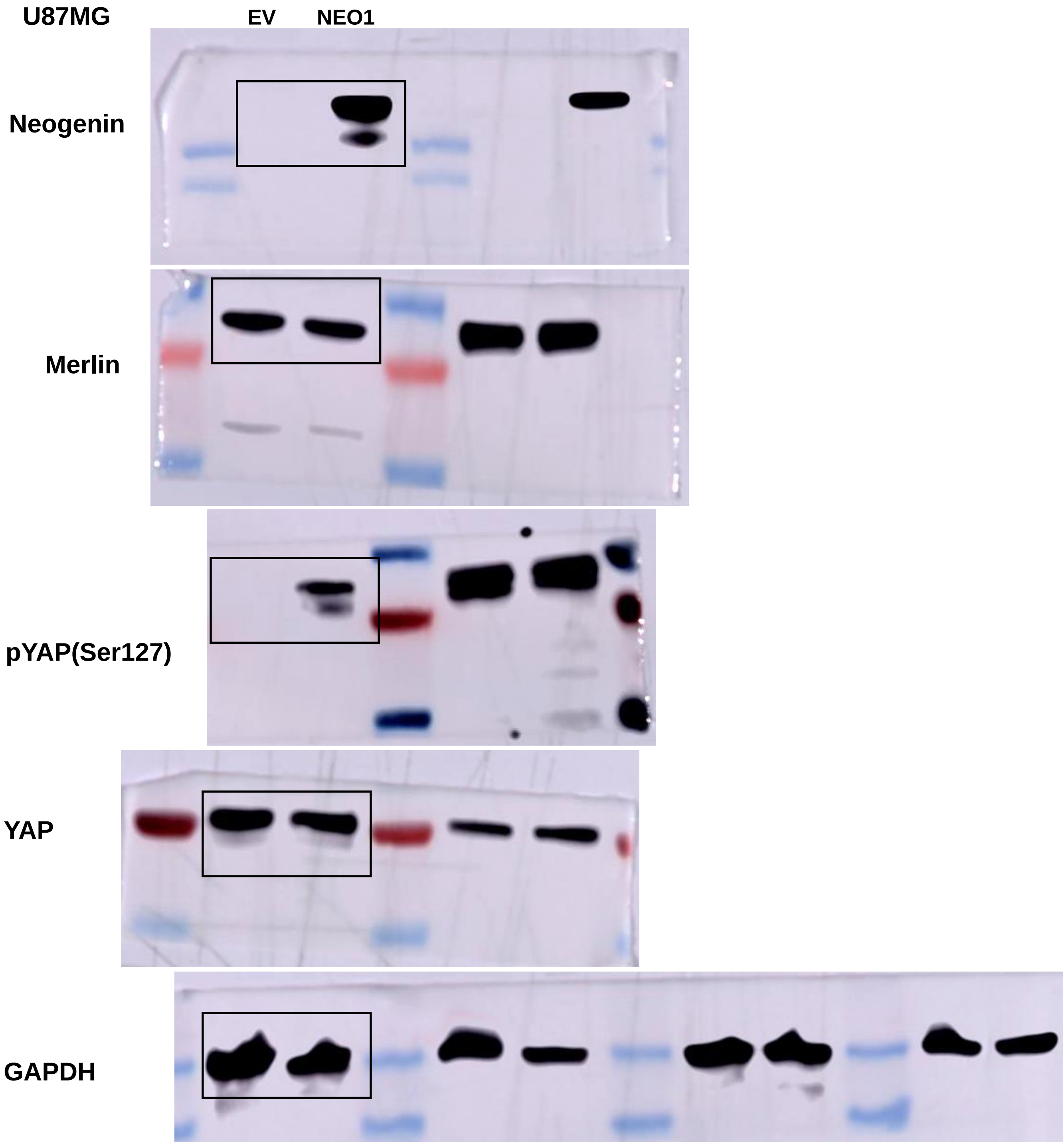

Fig.S7A

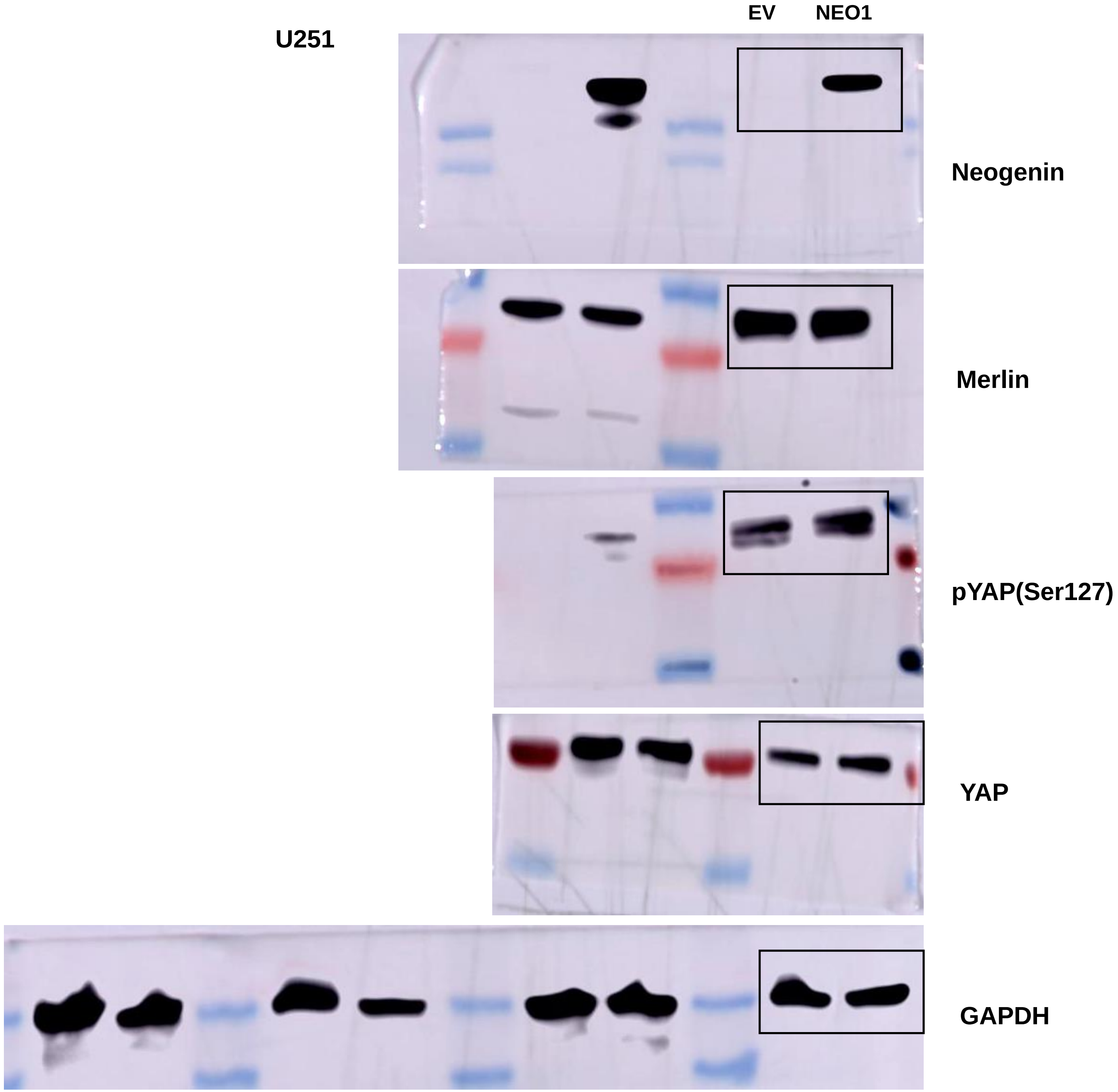

Fig.S7B

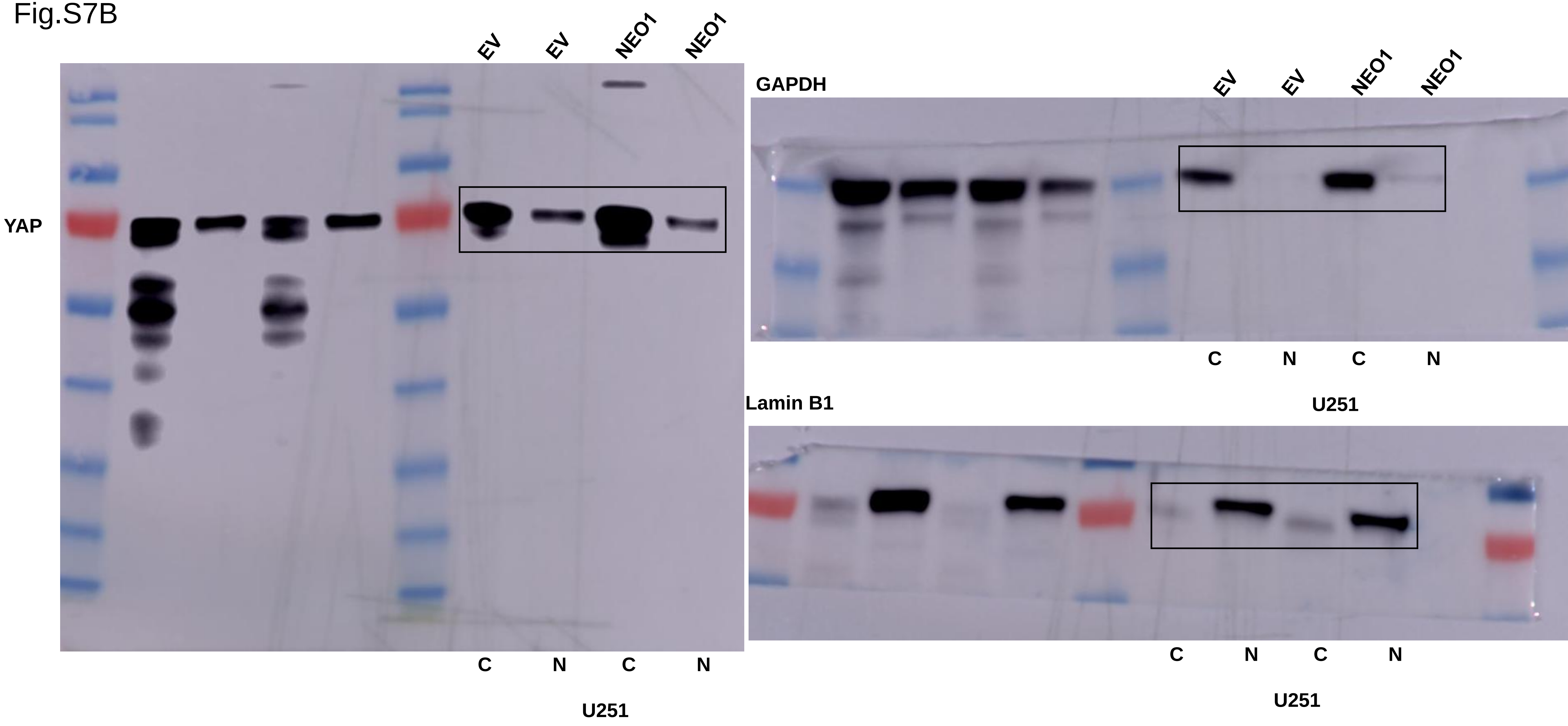

YAP
EV
EV
NEO1
NEO1
C
N
C
N
U251
GAPDH
EV
EV
NEO1
NEO1
C
N
C
N
U251
Lamin B1
C
N
C
N
U251

Fig.S7C

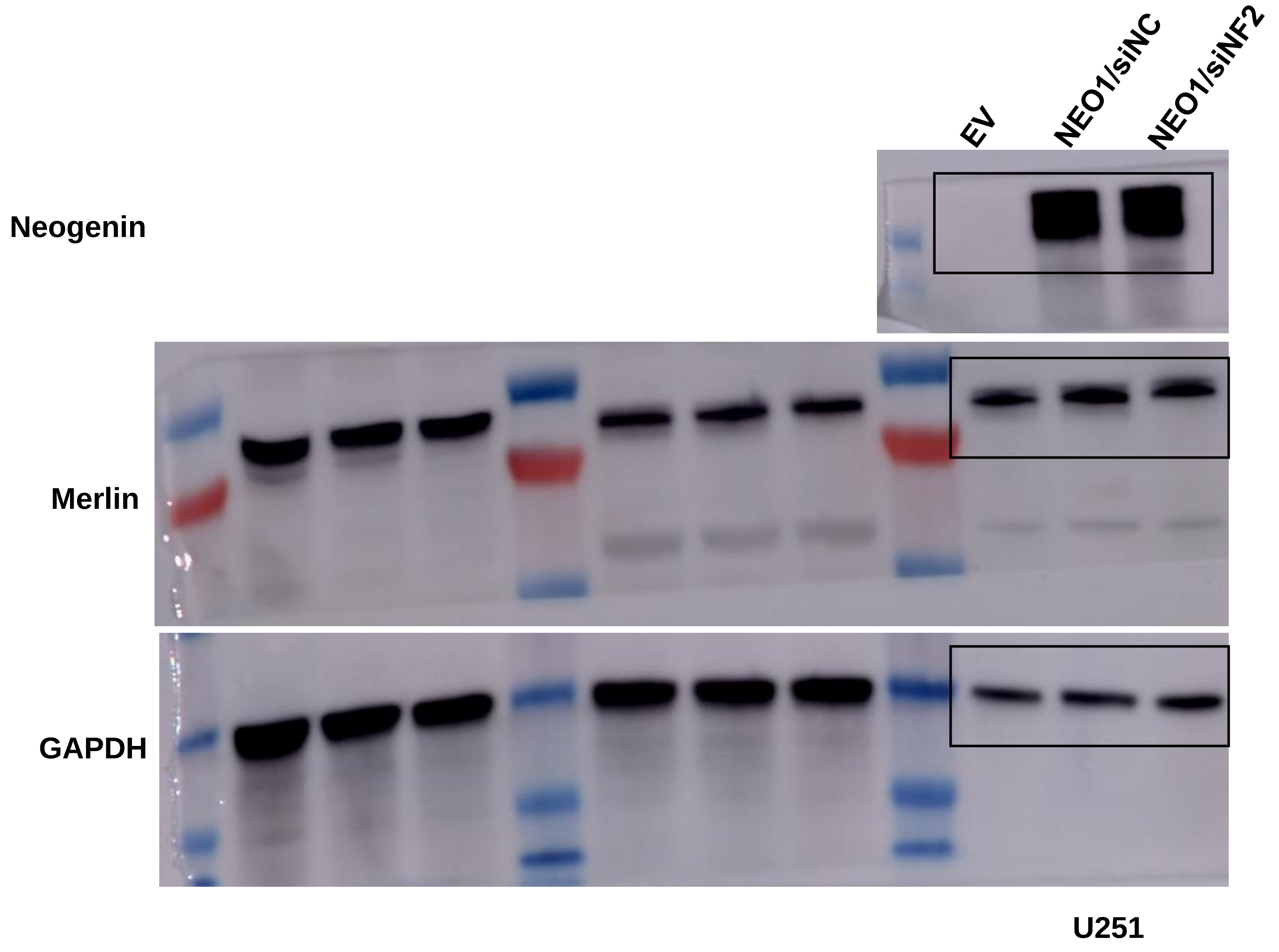

EV
NEO1/siNC
NEO1/siNF2
Neogenin
Merlin
GAPDH
U251

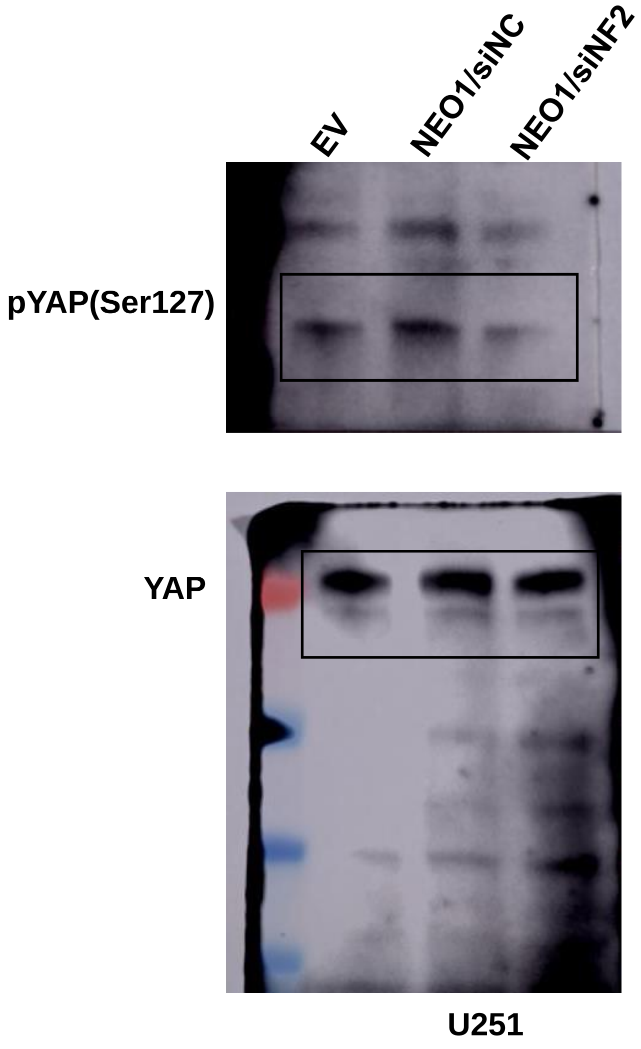

EV
NEO1/siNC
NEO1/siNF2
pYAP(Ser127)
YAP
U251
